# Naturally Derived Versus Synthetic Salicylic Acid: Physicochemical Properties, Transdermal Permeation and Macrophage Immunomodulation

**DOI:** 10.3390/antiox15101287

**Published:** 2026-10-03

**Authors:** Gamze Camlik, Ayça Altay Benetti, Gokhan Ozokan, Ulfet Erdogan Uzunoglu, Meltem Macit, Ebru Turkoz Acar, Cetin Tas, Ismail Tuncer Degim

**Affiliations:** 1 Department of Pharmaceutical Technology, Faculty of Pharmacy, Biruni University, Istanbul 34015, Türkiye; gcamlik@biruni.edu.tr (G.C.); tdegim@biruni.edu.tr (I.T.D.); 2 Department of Obstetrics and Gynaecology, National University of Singapore, Singapore 117597, Singapore; 3 BioArge Laboratories, Yildiz Technical University Technocity, Ikitelli, Istanbul 34490, Türkiye; gozokan@gmail.com; 4 Department of Chemistry and Biochemistry, Clarkson University, Potsdam, NY 13699, USA; erdoganu@clarkson.edu; 5 Department of Pharmaceutical Technology, Faculty of Pharmacy, Yeditepe University, Ataşehir, Istanbul 34755, Türkiye; meltem.macit@yeditepe.edu.tr (M.M.); cetin.tas@yeditepe.edu.tr (C.T.); 6 Department of Analytical Chemistry, Faculty of Pharmacy, Yeditepe University, Ataşehir, Istanbul 34755, Türkiye; ebru.acar@yeditepe.edu.tr

**Keywords:** salicylic acid, solid-state properties, transdermal permeation, dissolution, crystal engineering, extracellular vesicles, NanoFCM, RAW 264.7 macrophages, cytotoxicity, anti-inflammatory activity, immunomodulation

## Abstract

Salicylic acid is widely used in dermatological and cosmetic formulations, yet the influence of its production route on solid-state structure and biological behaviour remains underexplored. This study compares a naturally derived salicylic acid (NSA), obtained from wintergreen oil through green chemistry, with a synthetic form (SSA) produced via the Kolbe–Schmitt reaction, integrating physicochemical characterization with antioxidant and immunomodulatory profiling. Crystal morphology, crystallinity (XRD, FTIR), melting point, solubility, dissolution and Franz-cell skin permeation were determined, alongside intrinsic radical scavenging (DPPH), intracellular reactive oxygen species (DCFH-DA), cytoprotection (MTT), CD9^+^ extracellular-vesicle (EV) release and cytokine transcription in RAW 264.7 macrophages. SSA formed uniform, needle-like crystals of higher crystallinity, comparable equilibrium solubility (2.239 vs. 2.115 mg/mL; not significant) and faster dissolution, whereas the less-ordered NSA showed a numerically higher steady-state flux (39.94 ± 16.43 µg cm^−2^ h^−1^) and a shorter lag time (2.47 ± 1.71 h). SSA was the stronger radical scavenger (DPPH 20.7% vs. 8.7%) and reduced intracellular ROS more effectively, while NSA afforded greater cytoprotection. Both forms were associated with a lower CD9^+^ EV concentration in the conditioned medium following LPS stimulation, with SSA consistently more active across radical scavenging, ROS and vesicle endpoints. These findings show that the synthesis route, through its effect on solid-state properties, influences the physical performance, while the biological behaviour is discussed separately from the anti-inflammatory behaviour of salicylic acid, informing the rational selection of natural versus synthetic material for topical applications.

## 1. Introduction

Salicylic acid (SA), a β-hydroxy acid, is commonly used owing to its keratolytic, anti-inflammatory, antimicrobial, and comedolytic activities [1,2,3]. As a result of these characteristics, it is extensively applied in the management of conditions such as acne, psoriasis, and hyperkeratosis [3,4,5]. Moreover, it is widely employed in both topical and transdermal delivery systems [6,7]. Although salicylic acid has been widely used for decades, the efficacy of an active pharmaceutical ingredient (API) cannot be evaluated solely based on its chemical structure, as its performance is strongly influenced by solid-state properties [8,9]. It is well established that parameters such as crystal structure, morphology, polymorphism, and degree of crystallinity significantly affect key biopharmaceutical properties, including solubility, dissolution rate, and membrane permeability [10,11].

Recent studies have demonstrated that variations in solid-state characteristics can lead to notable differences in dissolution behaviour and drug performance, even when the chemical composition remains identical [10]. In particular, the amorphous form of a drug, due to its higher free energy and molecular disorder, often exhibits enhanced apparent solubility and dissolution compared to its crystalline counterpart [12].

In topical and transdermal drug delivery, these physicochemical properties are of critical importance, as drug absorption is governed not only by dissolution kinetics but also by diffusion across the *stratum corneum*, which acts as the primary barrier to permeation. Therefore, a comprehensive understanding of solid-state characteristics is essential for optimizing both drug release and membrane transport behaviour [13,14].

It is clear that properties such as crystal structure, morphology, and crystallinity can profoundly affect fundamental biopharmaceutical properties, including solubility, dissolution rate, and membrane permeability. In topical and transdermal drug delivery, these parameters are very important, as drug delivery depends on dissolution rate and diffusion through the *stratum corneum* [15,16]. Earlier research has demonstrated that differences in the crystal structure and lattice arrangement of a drug substance may alter the interfacial interactions between the drug and solvent and the mobility of molecules [17,18].

Even though SA shows low polymorphic diversity, variations in synthesis methods can lead to differences in crystallinity and structural uniformity [19]. SSA synthesized via the Kolbe–Schmitt reaction is notable for its uniform crystal lattice, achieved through well-controlled reaction conditions [20]. NSA, obtained from wintergreen-derived methyl salicylate through green-chemistry synthesis, may differ from SSA in crystal habit and preferred orientation. Such biological response can affect not only the physical properties of the two forms but also their biopharmaceutical behaviour [21].

In recent decades, considerable efforts have been made to investigate sustainable and natural pharmaceutical compounds [22]. Nevertheless, few studies have addressed the effect of synthesis-based differences in solid-state properties on both dissolution and skin permeability of these compounds [23,24]. Moreover, the interrelationship between the crystalline structure and the efficiency of SA penetration into the skin remains poorly understood [25].

Hence, the study aims to systematically compare natural and synthetic salicylic acid with respect to their solid-state properties and biopharmaceutical performance. The crystal structure and molecular architecture were elucidated by XRD and FTIR analysis. The solubility and dissolution properties were analyzed under physiological conditions. Moreover, transdermal permeation was examined using Franz diffusion cells with porcine skin as the membrane. It was hypothesized that variations in crystallinity and molecular architecture resulting from the synthesis process would lead to different dissolution and permeation results.

Beyond their physicochemical behaviour, the biological safety and immunomodulatory profile of a pharmaceutical active are decisive for its topical and transdermal use. The skin is continuously exposed to environmental insults, and it is now well established that oxidative stress and inflammation are closely interlinked in the pathogenesis of many cutaneous disorders, including acne, vitiligo, psoriasis and atopic dermatitis [26]. Reactive oxygen species (ROS) generated during these processes activate redox-sensitive transcription factors such as NF-κB, amplifying the inflammatory cascade [27]. Compounds that combine a favourable permeation profile with intrinsic antioxidant and anti-inflammatory activity are therefore of particular interest for dermatological and cosmetic formulations, which is directly relevant to salicylic acid, a monophenolic β-hydroxy acid already valued for its keratolytic and comedolytic properties [2]. On the other hand, macrophages are central effectors of cutaneous innate immunity, and the murine RAW 264.7 macrophage line is widely used to screen the cytotoxicity and anti-inflammatory activity of candidate compounds [28,29]. Because salicylic acid is frequently applied to compromised or inflamed skin, defining the concentration range over which it remains non-cytotoxic to such immune cells is an essential first step in establishing its biocompatibility, and any difference in cytotoxicity between natural and synthetic material could influence the therapeutic window of the final formulation [2].

Activated macrophages also release extracellular vesicles (EVs), nanometre-sized membrane particles that transport proteins, lipids and nucleic acids and serve as vehicles of intercellular inflammatory signalling. The tetraspanin CD9, together with CD63 and CD81, is a canonical EV and exosome surface marker and is particularly enriched on macrophage-derived vesicles where it contributes to membrane organization, vesicle biogenesis and immune communication; the output of CD9^+^ EVs therefore provides a sensitive readout of macrophage activation [30,31,32,33]. Anti-inflammatory agents can attenuate this activation, and salicylic acid (through its well-documented inhibition of NF-κB and cyclooxygenase signalling) might be expected to reduce the lipopolysaccharide (LPS)-driven release of CD9^+^ EVs from macrophages [33,34]. Whether natural and synthetic salicylic acid, which differ in solid-state properties and purity, also differ in this immunomodulatory capacity has not previously been reported. Accordingly, the present study extends the physicochemical comparison of NSA and SSA to a cellular context by evaluating their cytotoxicity in RAW 264.7 macrophages and their effect on CD9^+^ EV secretion, complemented by inflammatory gene-expression analysis. Accordingly, the present study extends the physicochemical comparison of NSA and SSA to a cellular context. We evaluate their cytotoxicity in RAW 264.7 macrophages, their intrinsic and cellular antioxidant activity (DPPH radical scavenging, H_2_O_2_-induced intracellular ROS and cytoprotection), and their effect on CD9^+^ EV secretion, complemented by inflammatory gene-expression analysis. By integrating solid-state characterization with antioxidant and immunomodulatory profiling, the study aims to establish how the synthesis route of salicylic acid shapes not only its biopharmaceutical performance but also its antioxidant and anti-inflammatory behaviour, thereby informing the rational selection of natural versus synthetic material for topical applications.

## 2. Materials and Methods

Sodium hydroxide and HCl were purchased from Sigma-Aldrich (St. Louis, MO, USA). All other chemicals were of analytical grade. Instruments used included an optical microscope (LV150N, Nikon Corporation, Tokyo, Japan) for morphological assessment, an X-ray diffractometer (MiniFlex 600, Rigaku Corporation, Tokyo, Japan) for crystallinity analysis, and a Fourier transform infrared spectrometer (QATR-S, Shimadzu Corporation, Kyoto, Japan) for structural characterization. Melting points were determined using an MPM-H2 melting point apparatus (LLG Labware, Meckenheim, Germany). Solubility measurements were carried out using a WiseBath shaking water bath (Daihan Scientific Co., Ltd., Wonju, Republic of Korea) and a Harmony Mixer Uzusio VTX-3000L (LMS Co., Ltd., Tokyo, Japan). Dissolution testing was performed using a USP Apparatus II (Pharma Test Apparatebau AG, Hainburg, Germany), and Franz diffusion cells (PharmaGear, Mumbai, India) with porcine skin were used for permeation studies.

### 2.1. Naturally Derived Salicylic Acid

**Figure 1:**
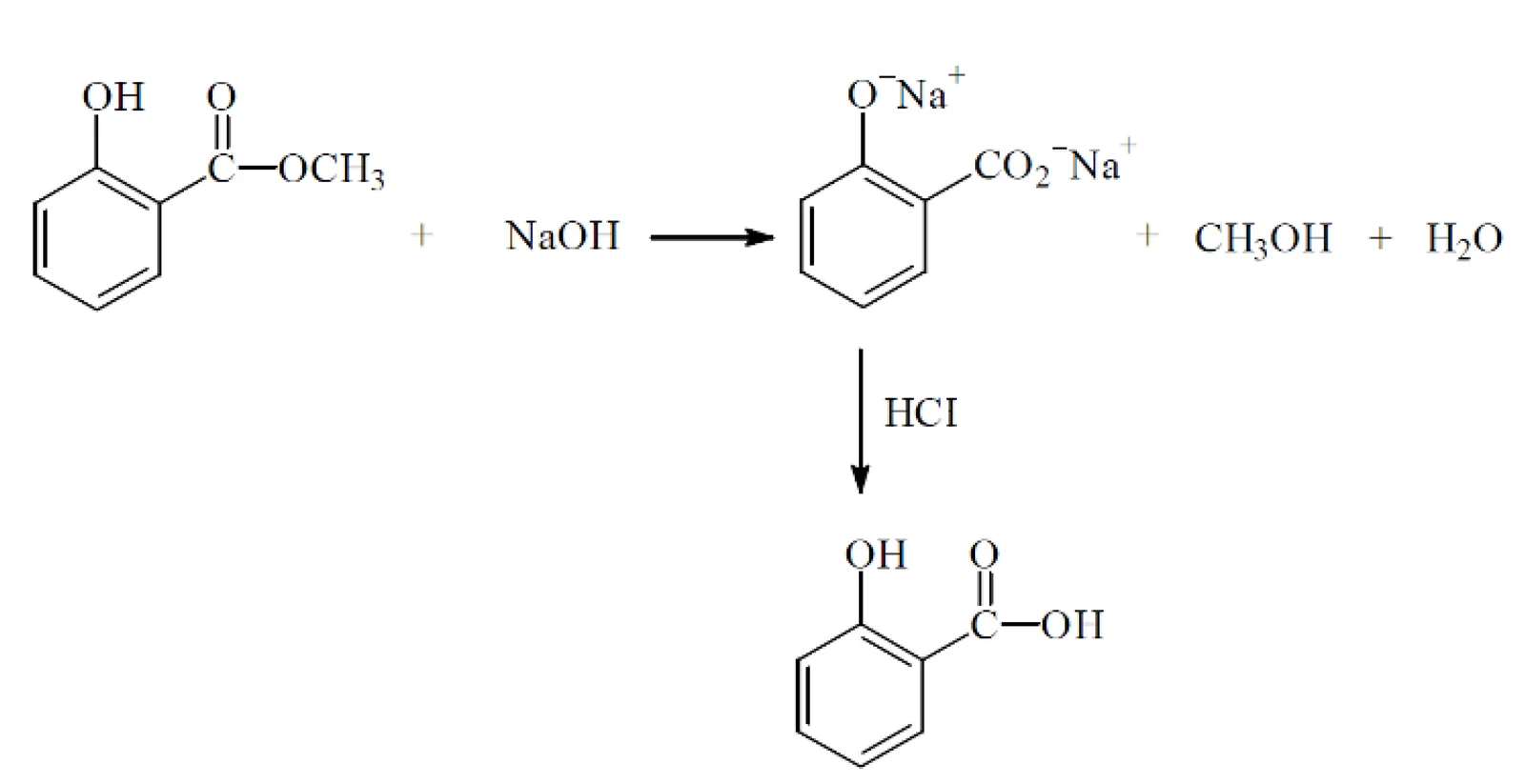
Production of naturally derived salicylic acid from wintergreen oil [20].

In total, 0.7 mol of 5 M sodium hydroxide solution is added to 0.1 mol of wintergreen oil over 10–15 min. White precipitates form. The heater and stirrer are turned on, and reflux is initiated. A homogeneous solution forms in 1 h, and reflux continues for a total of 3 h, with an additional 2 h of heating. After 3 h, the solution is cooled to room temperature. Then, 6 M HCl solution is slowly added at room temperature over 30 min to decrease the pH to 2–1.5. Heating occurs during this addition, and the solution is continuously cooled. Afterward, the solution is left to stand overnight, allowing more precipitate to form. Following this, it is filtered through a paper filter under vacuum. The crystals are washed with 100 mL of cold water and dried at 40 °C for 3–4 days Figure 1 [20].

### 2.2. Synthesis of Synthetic Salicylic Acid

**Figure 2:**
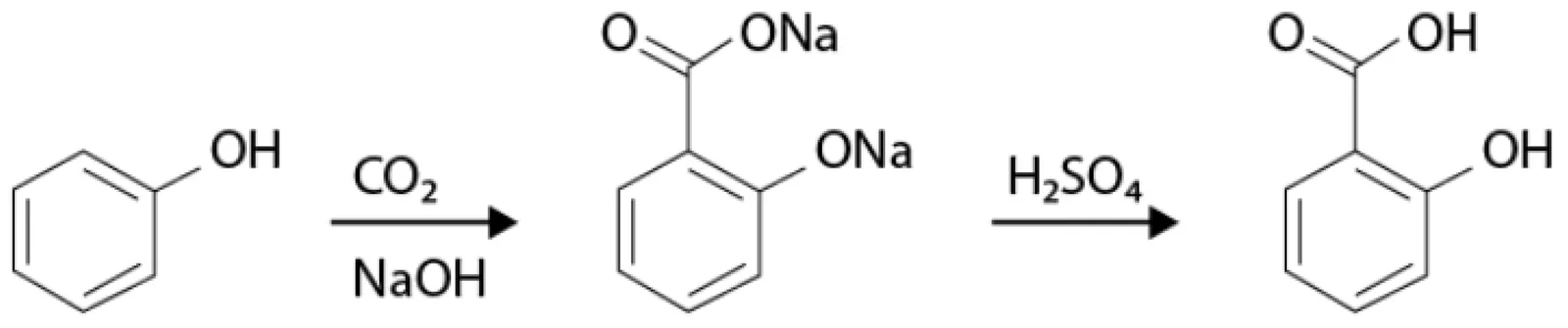
Synthetic salicylic acid was obtained by the Kolbe–Schmitt reaction [21].

Industrial Synthesis: Kolbe and Schmitt discovered the Kolbe–Schmitt reaction to synthesize salicylic acid from phenol, enabling large-scale commercial production (Figure 2). In this study, the synthetic salicylic acid (SSA) was synthesized in-house by this Kolbe–Schmitt route at BioArge Laboratories rather than obtained commercially.

Sodium phenoxide is heated with carbon dioxide at a pressure of 100 atmospheres and a temperature of 125 °C. The resulting product is then treated with sulfuric acid to produce salicylic acid [21]. Identity confirmed by ^1^H/^13^C NMR; purity by GC-MS (NSA ≥ 99%, SSA ≥ 93% area); see Supplementary (Figures S1–S3).

### 2.3. Images: Physical Appearance

Images of natural and synthetic salicylic acid were examined using an optical microscope (Nicon NV150N).

### 2.4. Melting Point Analysis

Melting is a physical process in which a substance changes from a solid to a liquid. This usually occurs when the internal energy of the solid increases due to heat or pressure, causing it to reach its melting point. The melting point is a key characteristic used to identify and distinguish crystalline solids. Pure substances have a narrow and well-defined melting range because of their regular crystalline lattice structure. In contrast, impurities disrupt the crystalline lattice and weaken intermolecular bonds. This causes melting to start at lower temperatures and results in a broader melting range [35]. The melting points of natural and synthetic salicylic acid were measured using the LLG/MPM-H2 device.

### 2.5. X-Ray Diffractometer (XRD) Analysis

XRD analysis (Rigaku MiniFlex 600 Powder X-ray Diffractometer, Tokyo, Japan) was used to examine the crystal structures of both natural and synthetic salicylic acid to determine whether they are crystalline or amorphous. Therefore, the influence of crystal structure on solubility and pharmaceutical performance was assessed [36]. XRD measurements were conducted on both NSA and SSA. XRD patterns were recorded over a 2θ range of 5–50° with a step size of 0.02° and a scanning rate of 2°/min. The instrument was operated at 40 kV and 30 mA.

### 2.6. Fourier Transform Infrared Spectroscopy (FTIR) Analysis

FTIR analyses (Shimadzu QATR-S, Shimadzu Corporation, Kyoto, Japan) were conducted to identify the chemical structure of natural and synthetic salicylic acid. FTIR was used to analyze NSA and SSA. Spectra were recorded over a wavenumber range of 4000–400 cm^−1^ with a resolution of 4 cm^−1^ using ATR mode.

### 2.7. Solubility Test

The solubility of natural and synthetic salicylic acid in both distilled water and pH 7.4 phosphate-buffered saline containing 25% ethanol was investigated. Solubility studies were conducted according to a published method [37]. An excess amount of pure SA (both natural and synthetic) was placed into each 3 mL microcentrifuge tube, to which 2 mL of distilled water and pH 7.4 phosphate-buffered saline containing 25% ethanol were separately added. The centrifuge tubes were vortexed for 6 min, then agitated in a thermostated shaking water bath at 25 °C ± 0.1 for 72 h at 50 rpm (Karl Kolb, Buchschlag, Germany). Afterward, the samples were centrifuged for 20 min at 20,000 rpm. The supernatant was then collected, diluted in the mobile phase, and filtered through a 0.45 μm membrane filter (Millipore Cellulose Acetate). Salicylic acid quantification was performed using an HPLC method; details are provided below.

The method for analyzing SA, as detailed in the 8th Edition of the European Pharmacopeia, has been utilized. Chromatographic separation was performed in isocratic mode. The mobile phase consisted of a mixture of glacial acetic acid, methanol, and water (1:40:60 *v*/*v*/*v*). The mobile phase solution was sonicated for 15 min, then filtered. A Zorbax^®^ C18 column (4.6 × 150 mm; 5 μm) was employed for separation. The injection volume was set at 10 µL, with a flow rate of 1 mL/min. Detection was performed at 270 nm, and the retention time was recorded at 7.2 min.

### 2.8. Dissolution Test

Dissolution studies of NSA and SSA were performed using USP Apparatus 2 (paddle method, PharmaTest) with three replicates. The dissolution medium consisted of 900 mL of phosphate buffer at pH 7.4, supplemented with 25% ethanol. The paddle was rotated at 50 rpm throughout the experiments. In each case, 5 mL of the dissolution sample was withdrawn at 5, 15, 30, 45, 60, 90, 120, 180, and 360 min, and an equal volume of fresh medium was added to maintain the volume constant. Samples were analyzed by HPLC as previously described. The cumulative percentage of drug dissolved was calculated using calibration equations.

### 2.9. Permeation Through the Skin Analysis

Pig skin obtained from the dorsal (back) region of the animal was used for the in vitro permeation studies. Hair and subcutaneous adipose tissue were removed using surgical scissors to yield a skin slice as described in previous studies [38,39,40,41]. The prepared skin samples were sealed and stored at −20 °C until use. Prior to the permeation experiments, the skin samples were thawed at room temperature and washed with distilled water. Skin integrity was visually assessed using a stereomicroscope (Carl Zeiss, Stemi DV4, Germany) [42]. The skin samples were then mounted in open, modified Franz diffusion cells with an available diffusion area of 4.91 cm^2^ and a receptor volume of approximately 20 mL. Permeation used skin from three independent donors (n = 3), one Franz cell per donör and sink conditions were maintained throughout the study [42]. The receptor side of the diffusion cells was maintained at 37 °C by circulating thermostated water. The linear portion of the permeation curve was used to calculate the steady-state flux by linear regression analysis. The receptor compartments contained phosphate-buffered solution of pH 7.4 with 25% ethanol and were stirred with a Teflon-coated magnetic bar. A saturated solution of both natural and synthetic salicylic acid in distilled water (3 mL) was applied to the stratum corneum side of the porcine skin membrane. At appropriate time intervals (5, 10, 15, 30, 45, 60, 75, 90, 120, 150, 180, 210, 240, 300, 360, 420, 480, 720, 1440 min), receptor fluid (0.8 mL) was withdrawn and replaced with fresh, thermostated receptor fluid. It was ensured that sink conditions were maintained and that the sample concentrations exceeded the limit of quantitation of the analytical methods. The experiments were performed in triplicate at minimum. Based on the obtained concentrations and the amount of receptor fluid withdrawn at each time, the permeated cumulative amount of drug was calculated for each diffusion cell and plotted over time. For all individual cells, flux was determined from the slope of the linear part of the curve, and lag times were identified as the x-intercept, using linear regression analysis.

### 2.10. Cell Culture

RAW 264.7 murine macrophages were maintained in Dulbecco’s Modified Eagle Medium (DMEM) supplemented with 10% fetal bovine serum (FBS) and 1% penicillin–streptomycin at 37 °C in a humidified atmosphere containing 5% CO_2_ [43]. For extracellular-vesicle (EV) experiments, FBS was omitted from the culture medium and cells were maintained in serum-free DMEM (with 1% penicillin–streptomycin); serum was deliberately excluded so that bovine serum-derived EVs could not contaminate the macrophage-derived EV preparations. Cell morphology was documented by phase-contrast microscopy (×20 objective).

### 2.11. Cytotoxicity (MTT) Assay

Cytotoxicity was assessed using the MTT assay. RAW 264.7 cells were seeded into 96-well plates (1 × 10^4^ cells/well) and allowed to adhere for 24 h. Cells were then treated for 24 h with NSA or SSA across a concentration range of 1–1000 µg/mL. After treatment, MTT reagent (final 0.5 mg/mL) was added, and the plates were incubated for 3–4 h at 37 °C. The resulting formazan crystals were dissolved in dimethyl sulfoxide, and the absorbance was measured at 570 nm. Cell viability was expressed as a percentage of untreated control cells, and half-maximal inhibitory concentrations (IC_50_) were derived by nonlinear regression ([inhibitor] vs. normalized response, variable slope) using GraphPad Prism 11.0.

### 2.12. Extracellular-Vesicle Isolation

EVs were isolated from macrophage-conditioned medium by size-exclusion chromatography using an Izon qEV column (EV1 Original series) according to the manufacturer’s instructions. Conditioned medium was collected from untreated (control), LPS-stimulated (100 ng/mL), and LPS + salicylic acid–treated cells, pre-cleared by low-speed centrifugation to remove cells and debris and applied to the pre-equilibrated column. EV-enriched fractions were eluted with phosphate-buffered saline and pooled for analysis.

### 2.13. NanoFCM Analysis of CD9^+^ EVs

Isolated EVs were immunolabelled for the tetraspanin CD9^+^ using the ExoBrite CD9^+^ (Biotium, Fremont, CA, USA) probe/kit and analyzed by nano-flow cytometry (NanoFCM, USA) at the single-particle level. Particle size and concentration were calibrated against silica nanosphere size standards and a concentration reference standard; a particle-free buffer blank was included to confirm background specificity. Results are reported as median and mean particle diameter and as particle concentration (particles/mL). In addition, the total protein content of the isolated EV fractions was determined spectrophotometrically at 280 nm (DeNovix, USA).

### 2.14. Anti-Inflammatory Gene Expression (RT-qPCR)

Total RNA was extracted from treated RAW 264.7 macrophages, and its concentration and purity were assessed spectrophotometrically (DeNovix, USA). RNA was reverse-transcribed into cDNA, and the expression of the pro-inflammatory cytokine genes Il1b (IL-1β) and Tnf (TNF-α) was quantified by real-time PCR using GAPDH as the housekeeping reference gene. All treatment groups (LPS, Dex + LPS, SSA + LPS and NSA + LPS) were co-stimulated with LPS (100 ng/mL). Relative gene expression was calculated by the 2^−ΔΔCt^ method using untreated cells as the calibrator, and results are reported from independent biological replicates (n = 3).

### 2.15. DPPH Radical-Scavenging Assay

The intrinsic radical-scavenging activity of the two salicylic acid forms was determined using a cell-free 2,2-diphenyl-1-picrylhydrazyl (DPPH) assay. Solutions of NSA and SSA (20 µg/mL) and ascorbic acid (25 µM, positive control) were mixed with a methanolic DPPH solution and incubated in the dark at room temperature. The decrease in absorbance at 517 nm was recorded against a methanol/DPPH blank, and radical-scavenging activity was expressed as the percentage reduction in absorbance relative to the DPPH control. Measurements were performed in triplicate. Ascorbic acid served as a qualitative positive control; because the salicylic acids (20 µg/mL, 145 µM) and ascorbic acid (25 µM) were not tested at equimolar concentrations, no direct potency comparison was made between them.

### 2.16. Intracellular Reactive Oxygen Species (DCFH-DA)

Intracellular reactive oxygen species (ROS) were measured in RAW 264.7 macrophages using the fluorogenic probe 2′,7′-dichlorodihydrofluorescein diacetate (DCFH-DA, Sigma-Aldrich, USA). Cells were exposed to 100 µM H_2_O_2_ for 1 h to induce oxidative stress, either alone or together with NSA or SSA (20 µg/mL) or ascorbic acid (25 µM). After incubation, DCF fluorescence was recorded and expressed relative to untreated and H_2_O_2_-only controls. Measurements were performed in triplicate.

### 2.17. Cytoprotection Against Oxidative Injury

Protection of cell viability under oxidative challenge was assessed in parallel by the MTT assay. RAW 264.7 macrophages were exposed to 100 µM H_2_O_2_ for 1 h, alone or with NSA or SSA (20 µg/mL) or ascorbic acid (25 µM), and viability was expressed as a percentage of the untreated control. A single sub-cytotoxic concentration (20 µg/mL) was used for all salicylic acid treatments, matching that of the extracellular-vesicle and RT-qPCR experiments, so that the antioxidant, transcriptional and vesicle readouts could be compared under identical conditions. Data are expressed as mean ± SD and compared by two-sample *t*-tests.

### 2.18. Statistical Analysis

All experiments were performed in triplicate (n = 3) unless otherwise stated. Data are presented as mean ± standard deviation (SD). Statistical analysis was performed using one-way analysis of variance (ANOVA) followed by Student’s *t*-test for pairwise comparisons between NSA and SSA. Differences were considered statistically significant at *p* < 0.05.

## 3. Results

### 3.1. Naturally Derived Salicylic Acid (NSA)

Green synthesis of NSA from wintergreen oil was successfully performed to give crystalline material with a yield above 95%. The microscopic analysis reveals an irregular crystal habit, varying from cubical to orthorhombic. The diversity in crystal structure is an indication that there is no uniform structure compared to SSA.

The irregular crystal habit of NSA could indicate a less-ordered structure, reducing molecular packing and potentially contributing to solubility [44]. In addition, structural variation and possible contaminants associated with the natural synthesis process may also contribute to the differences in the physicochemical properties of NSA [45].

In conclusion, the green chemical synthesis method influences the crystal structure and provides pharmaceutical properties different from those of synthetic methods [46].

### 3.2. Synthetic Salicylic Acid (SSA)

Needle-shaped crystals with uniform size and shape were observed for SSA synthesized using the Kolbe–Schmitt reaction. This shows that the structural ordering was high, since the product showed uniform crystals.

The ordered nature of SSA implies that the molecular arrangement in the crystal structure is highly effective. This ordered nature would improve its biopharmaceutical activity relative to the natural product, leading to uniform dissolution and diffusion [47].

### 3.3. Physical Appearance

**Figure 3:**
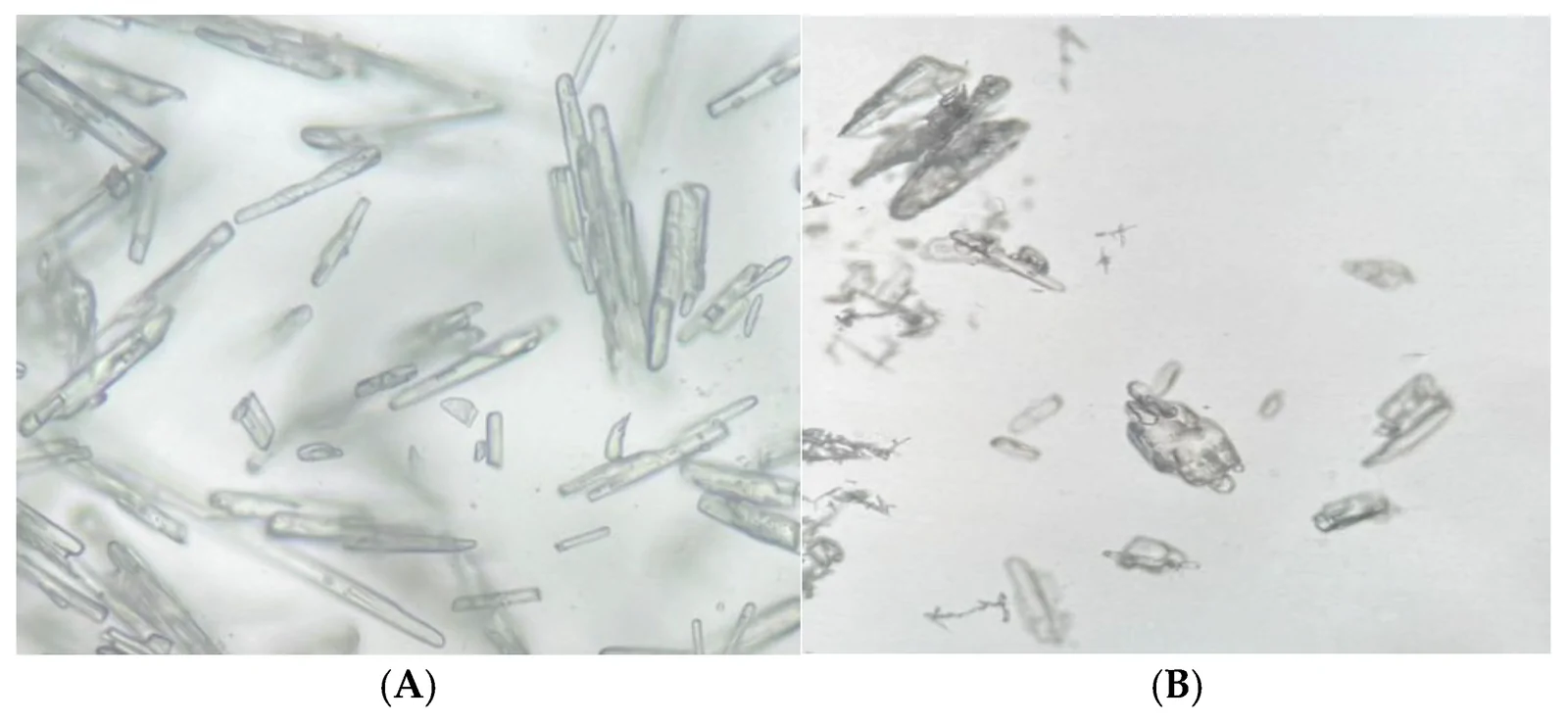
(**A**) microscopic image of NSA; (**B**) microscopic image of SSA (10× objective lens).

Analysis under a microscope Figure 3 revealed morphological differences between the NSA and SSA crystals. While the NSA crystals were fragmented irregular prisms, the SSA crystals were elongated needles. These differences in crystal habit can arise from different crystallization conditions. The more regular, needle-like morphology of SSA is consistent with a strong preferred orientation, whereas the more heterogeneous habit of NSA reflects a less uniform crystal growth [46,47,48].

### 3.4. Melting Point

The melting points of NSA and SSA were 158.2 °C and 160.5 °C, respectively. The two melting points are quite close to each other; however, this small difference is not by itself indicative of a difference in purity, since the identity and high purity of both forms were confirmed by chromatographic and spectroscopic analysis (Section 2). The slightly lower melting point of NSA may reflect differences in crystal habit or lattice order rather than a difference in purity, resulting in a broader melting range than that of SSA [49,50].

### 3.5. X-Ray Diffractometer (XRD)

**Figure 4:**
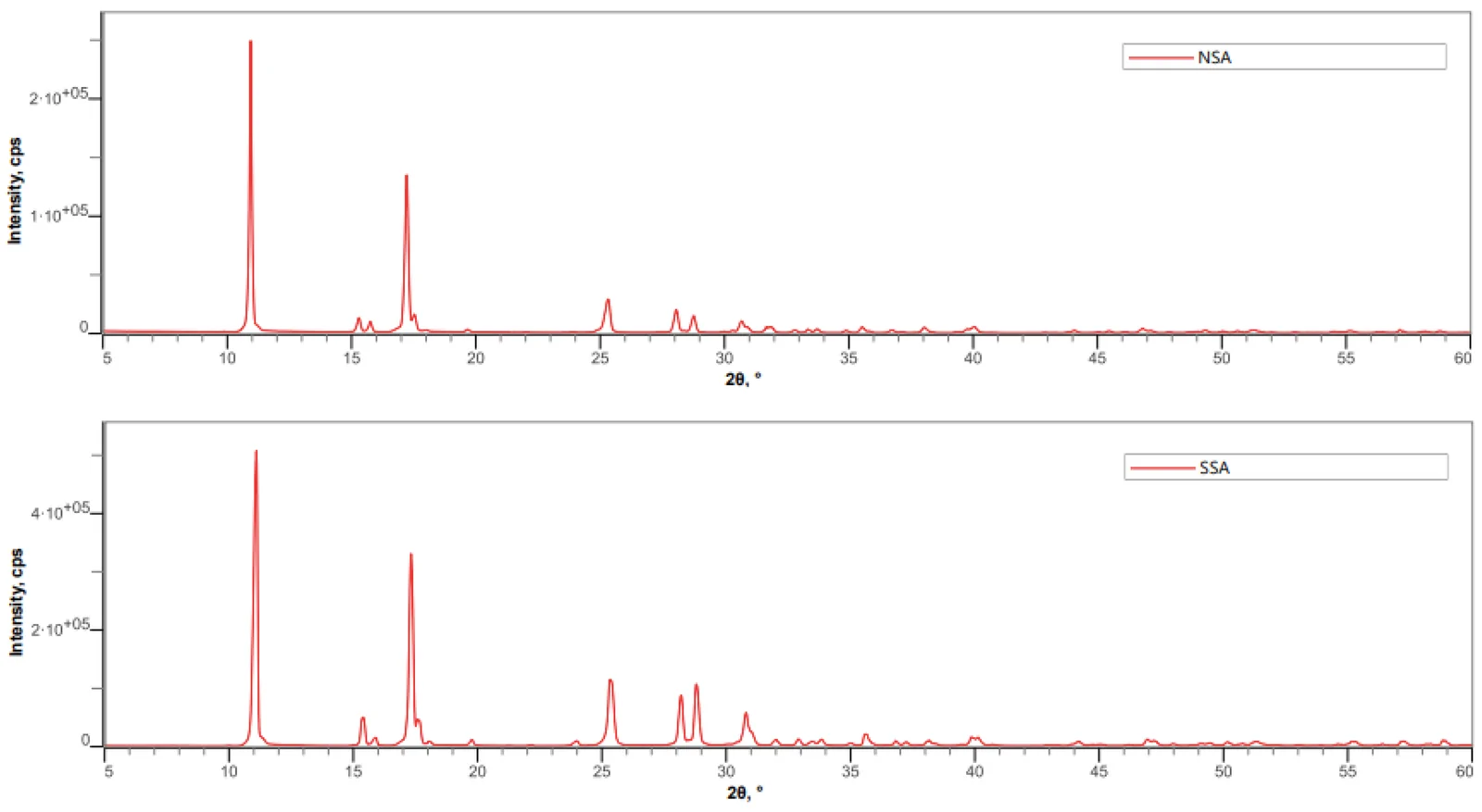
X-Ray Diffractometer measurement results of NSA and SSA.

The XRD results in Figure 4 show that both NSA and SSA have crystalline structures but with substantial differences in the shape of the peaks. Both patterns display sharp, well-defined reflections, confirming that the two forms are crystalline rather than amorphous. The experimental NSA pattern closely matches the powder pattern simulated from the published single-crystal structure of salicylic acid (CSD refcode SALIAC; Cochran, 1953) [51], with reflections at 2θ ≈ 11.1°, 17.3°, 25.4°, 28.6° and 30.8° corresponding to the reference positions, confirming the identity and crystalline nature of the material. The SSA pattern shows the same family of reflections but with a markedly different relative-intensity distribution and a dominant low-angle reflection at 2θ ≈ 11.6°, consistent with strong preferred orientation arising from its uniform needle-like crystal habit rather than with a change in phase.

Minor additional low-intensity reflections in the NSA pattern may reflect subtle differences in crystallite size or sample texture between the two preparations [52]. The distinct crystal habit and texture of the two forms, rather than a difference between crystalline and amorphous states, are considered relevant to their dissolution and permeation behaviour [53].

### 3.6. Fourier Transform Infrared Spectroscopy (FTIR)

**Figure 5:**
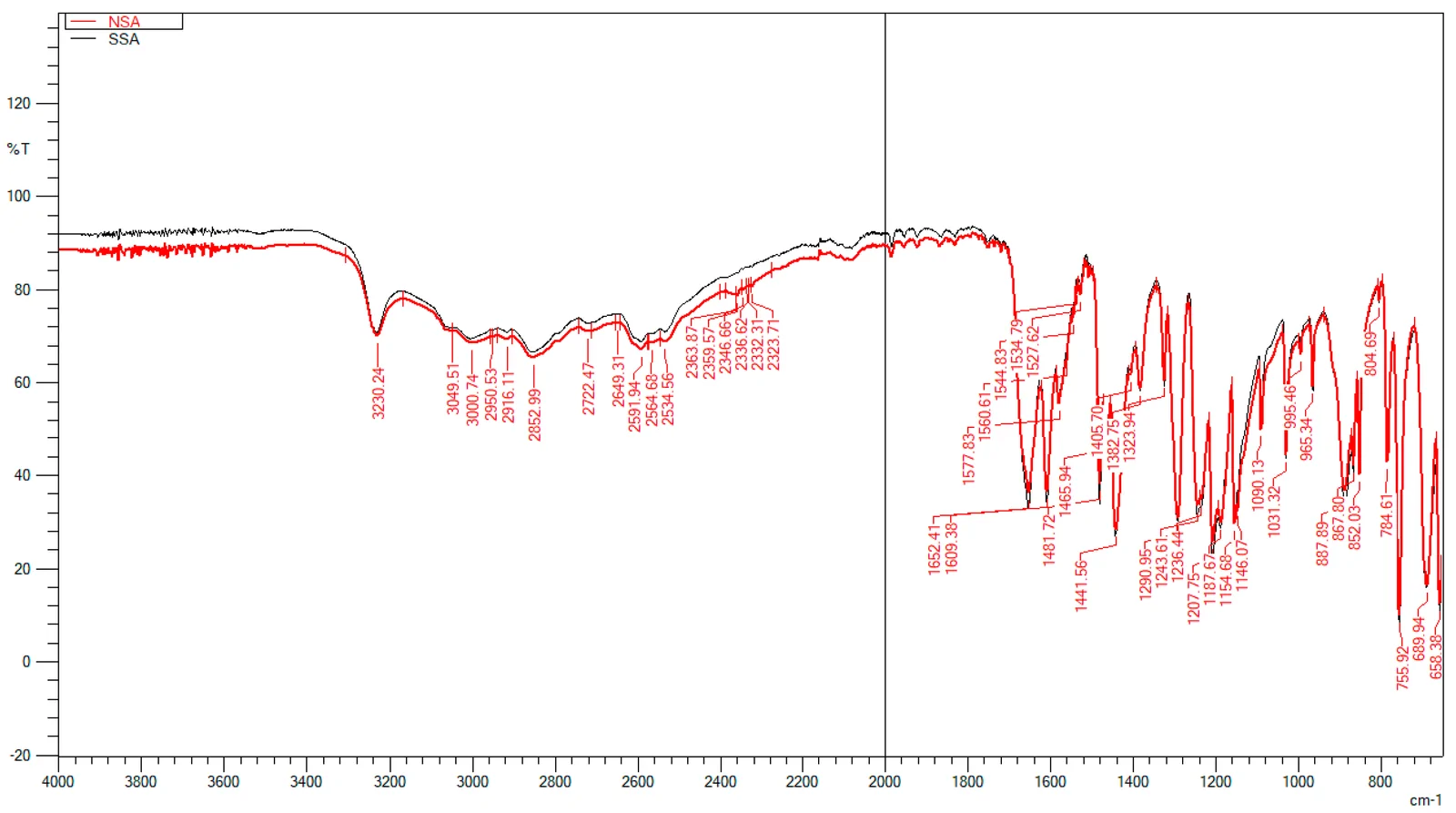
FTIR results of NSA and SSA.

The FTIR spectra of NSA and SSA showed that the samples had typical functional groups of salicylic acid Figure 5. However, the spectra of SSA displayed sharper peaks than those of NSA, suggesting higher orderliness in the molecular arrangement.

Conversely, the spectra of NSA showed peak broadening and slight shifts, primarily in the C-O region, suggesting the presence of different structures and intermolecular interactions. The observed variations could be attributed to changes in hydrogen-bonding characteristics.

### 3.7. Dissolution

**Figure 6:**
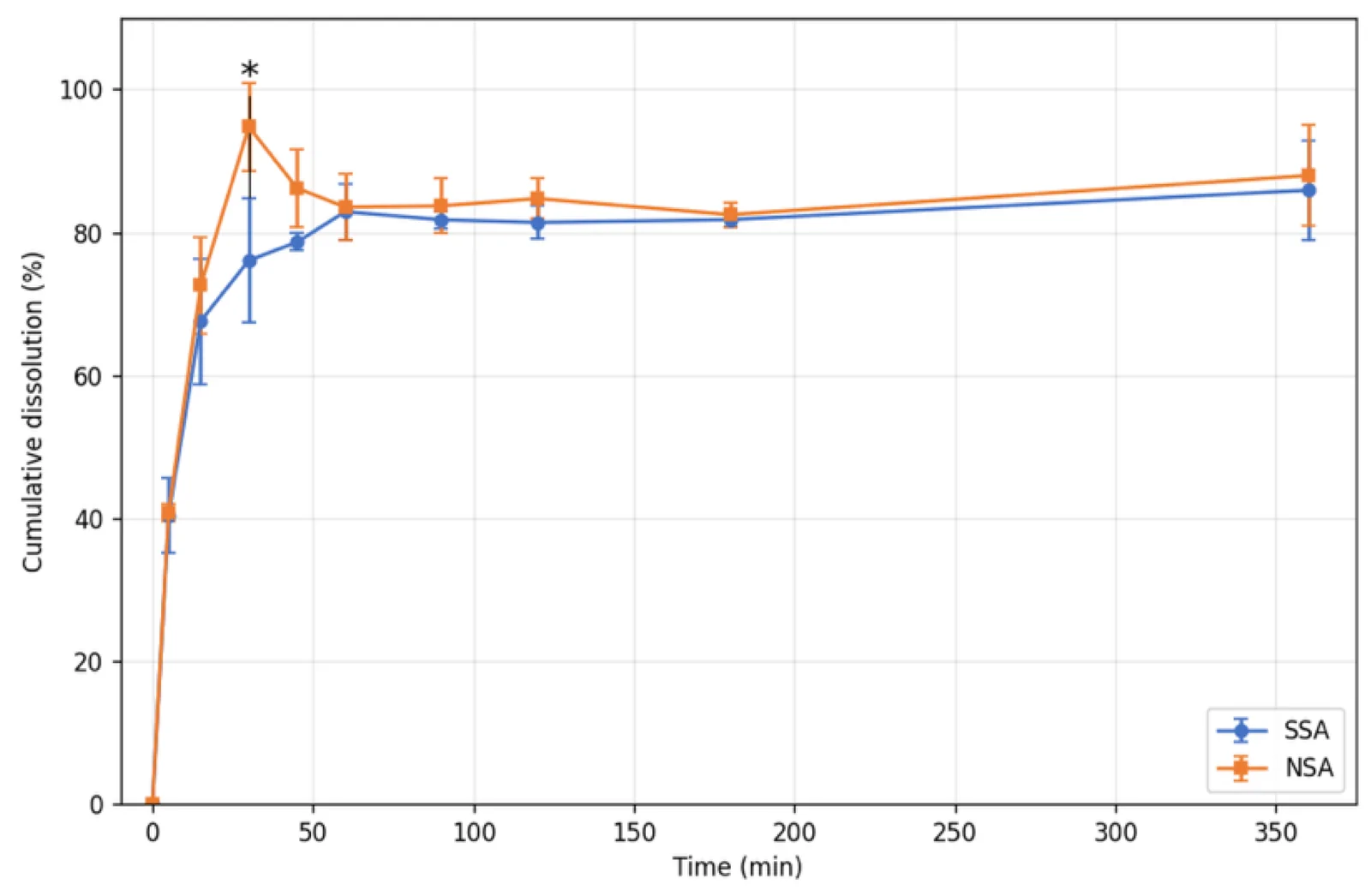
NSA and SSA of percentage solubility (Data are presented as mean ± SD (n = 3)).

The dissolution profiles of NSA and SSA were studied using the USP Apparatus II (paddle method) in a pH 7.4 phosphate buffer containing 25% ethanol Figure 6. Both forms showed rapid dissolution during the first 15 min, but NSA reached a significantly higher initial dissolution rate compared to SSA (*p* < 0.05). Minor fluctuations were observed for NSA during the early phase, and after 60 min the dissolution profiles of both forms became similar and stabilized at 85–90%. No significant difference was detected in long-term evaluations (180–360 min).

The rapid initial dissolution of SSA is most likely related to its uniform, needle-like crystal habit, which may allow more homogeneous contact with the dissolution medium [54]. Differences in crystallite size and crystal habit between the two forms, rather than a difference between crystalline and amorphous states, may account for the slower early dissolution of SSA compared to NSA [55]. On the other hand, it should be noted that particle-size distribution and specific surface area, which strongly influence dissolution, were not measured here and may also contribute to this difference.

The findings indicate that crystal structure and morphology are decisive factors in dissolution behaviour. While NSA offers an advantage for formulations requiring rapid-onset release, the fact that both forms exhibit similar dissolution profiles over time suggests that these biological responses have a diminishing effect on long-term performance [56].

### 3.8. Solubility

The solubilities of NSA and SSA were compared in pure water and pH 7.4 phosphate buffer with 25% ethanol. In both forms, solubility in the ethanol-containing medium was significantly higher. The average solubility was 2.239 mg/mL for SSA and 2.115 mg/mL for NSA; however, this difference was not statistically significant (*p* > 0.05). These values were approximately doubled in the ethanol-containing buffer medium Table 1.

Because this difference in solubility was not statistically significant, the two forms are considered to have comparable equilibrium solubility; any minor numerical difference cannot be reliably attributed to differences in crystal structure [10]. Any small differences in dissolution behaviour are therefore more likely related to crystal habit and particle characteristics than to a genuine difference in equilibrium solubility [57].

The composition of the medium was also found to be a determining factor in solubility. The solubility values obtained from HPLC analyses are shown in Table 1. The presence of ethanol increased the solubility of both forms by weakening hydrogen bonds and altering the solvent’s dielectric properties. This indicates that solubility behaviour is closely related not only to crystal structure but also to the properties of the medium.

In conclusion, the two forms exhibit comparable equilibrium solubility, so neither can be recommended over the other on the basis of solubility alone. The choice between them is more appropriately guided by other properties, such as skin permeation behaviour and the intended route of administration.

### 3.9. Permeation Through the Skin

**Figure 7:**
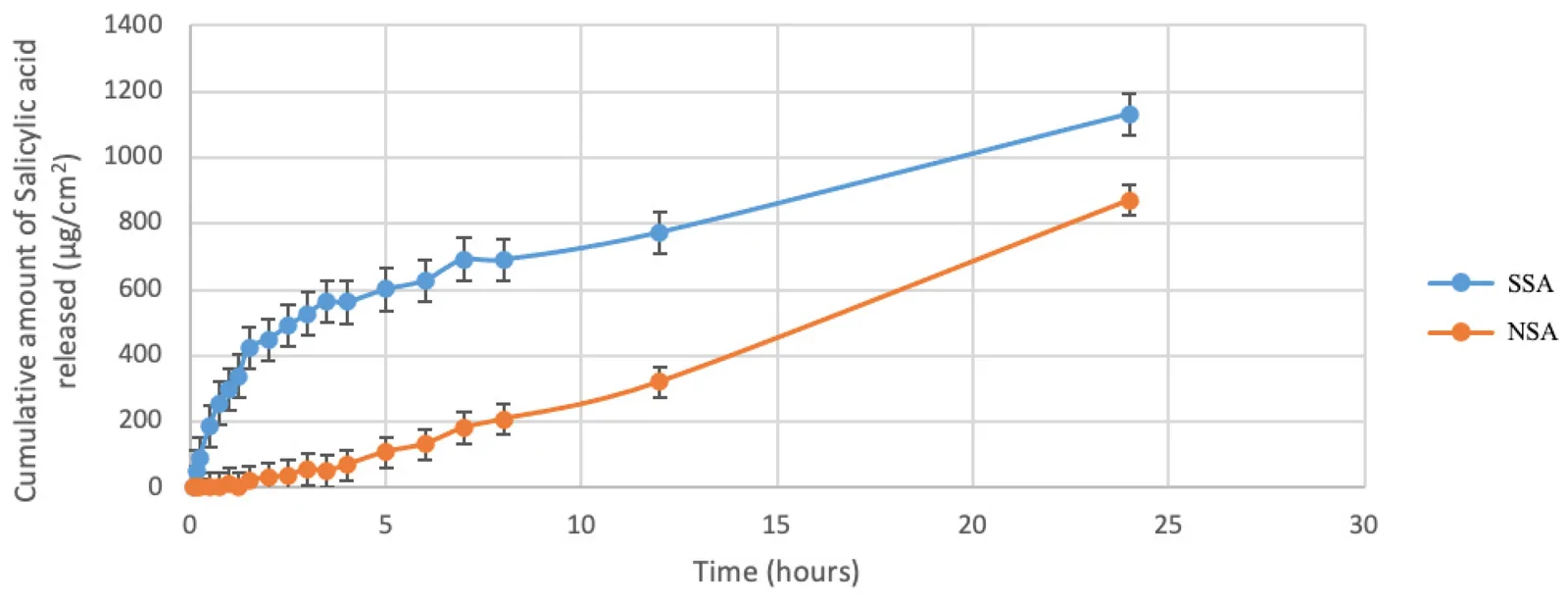
Permeation test results of NSA and SSA (Data are presented as mean ± SD (n = 3)).

In the Franz diffusion cell study, saturated solutions of both natural and synthetic salicylic acid were applied to the porcine skin surface, and the permeation test results are presented in Figure 7 and Table 2.

The in vitro permeation study revealed differences in the permeation profiles of NSA and SSA. As illustrated in Figure 7 below, the cumulative amount of SSA permeated exceeded 1200 µg/cm^2^ in 24 h, while NSA attained 800 µg/cm^2^ over the same period.

NSA exhibited a numerically higher steady-state flux (Jss) than SSA, with values of 39.94 ± 16.43 and 35.57 ± 13.91 µg cm^−2^ h^−1^, respectively. Similarly, the permeability coefficient was numerically higher for NSA (15.98 ± 6.57 × 10^−3^ cm h^−1^) than for SSA (14.23 ± 5.56 × 10^−3^ cm h^−1^). NSA also showed a shorter lag time (2.47 ± 1.71 h) compared with SSA (3.28 ± 0.55 h). Time-dependent permeation profiles showed variation in the permeation rate of the two forms over the first 8 h, with SSA showing higher permeation rates. However, beyond the tenth hour, the cumulative permeation rate of NSA increased markedly.

These findings indicate that, despite the higher cumulative permeation observed for SSA, NSA showed a tendency toward higher steady-state flux and permeability coefficient together with a shorter lag time. The observed differences may be associated with variations in crystal structure and physicochemical properties [58,59,60].

### 3.10. Cytotoxicity of NSA and SSA in Macrophages

**Figure 8:**
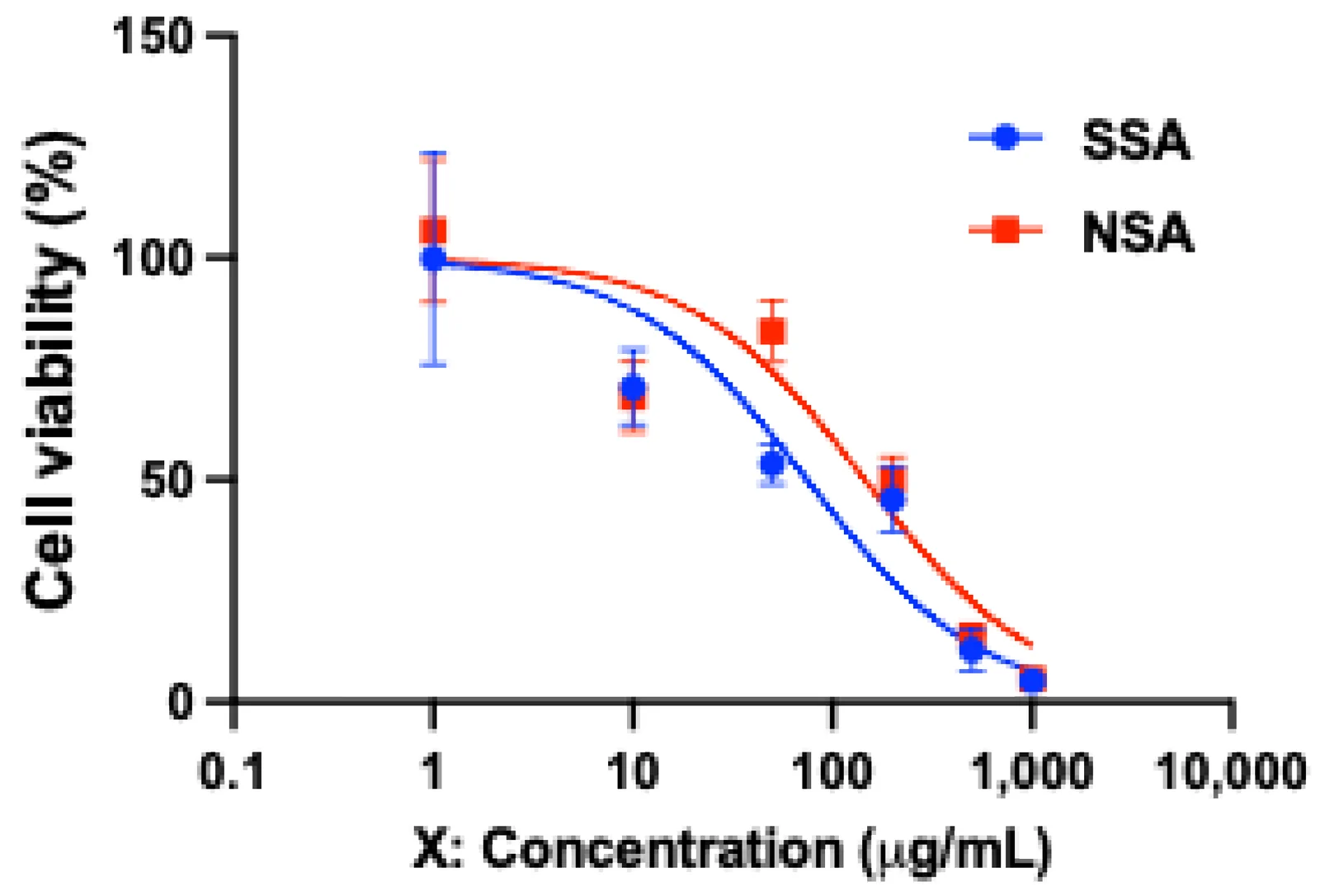
MTT dose–response of NSA and SSA in RAW 264.7 macrophages (mean ± SD). Curves show variable-slope nonlinear regression fits; IC_50_ values were determined in GraphPad Prism.

Both salicylic acids reduced RAW 264.7 macrophage viability in a concentration-dependent manner Figure 8. Nonlinear regression yielded IC_50_ values of 74.7 µg/mL (95% CI 31.3–161.7; R^2^ = 0.80) for SSA and 145.6 µg/mL (95% CI 75.2–266.0; R^2^ = 0.84) for NSA, indicating that NSA was tolerated at approximately two-fold higher concentrations than SSA. At low concentrations (≤10 µg/mL), viability remained close to control values for both compounds, confirming a sub-cytotoxic working range for the subsequent EV experiments.

### 3.11. Macrophage Morphology

**Figure 9:**
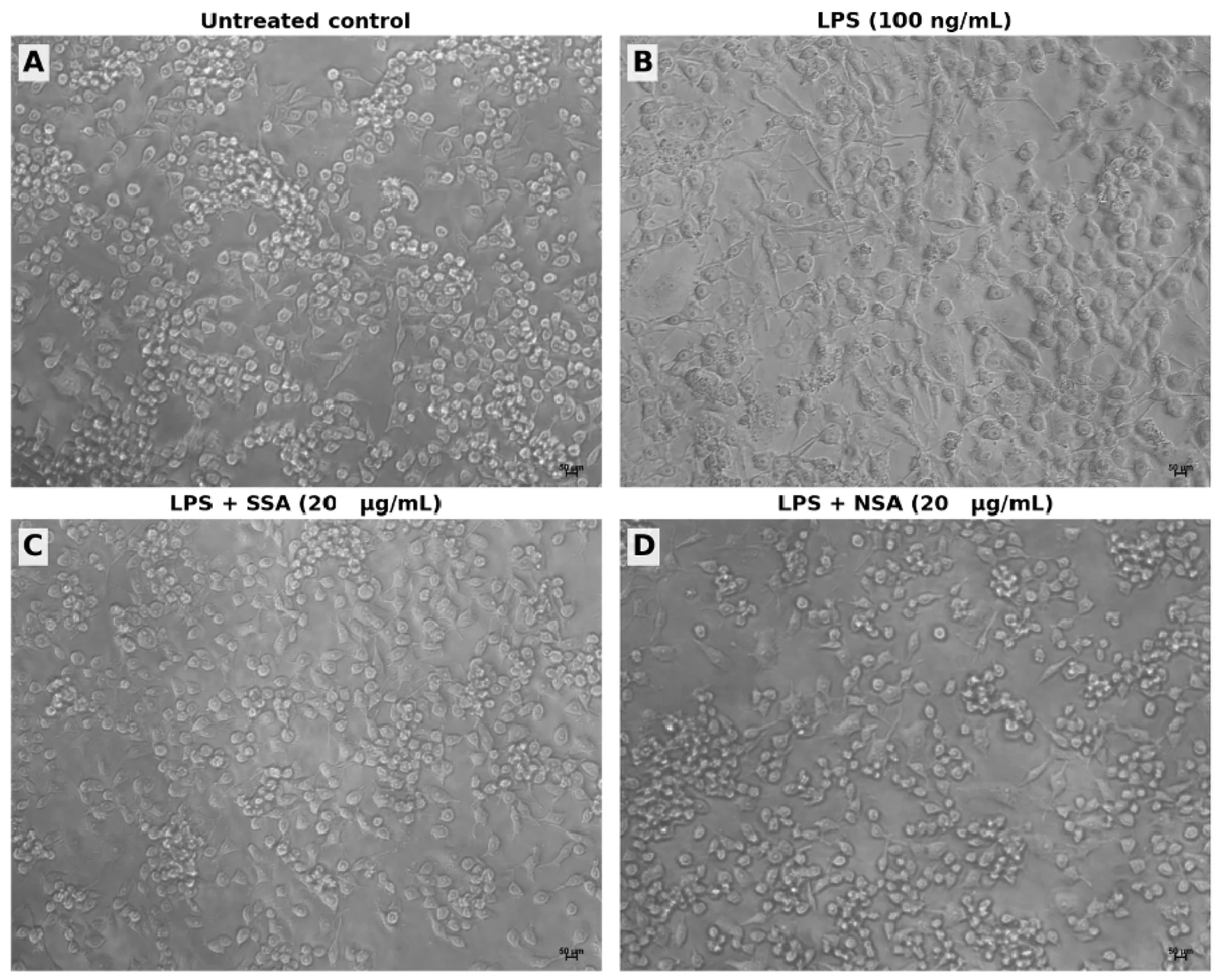
Representative phase-contrast micrographs (×20) of RAW 264.7 macrophages: (**A**) untreated control, (**B**) LPS (100 ng/mL), (**C**) LPS + SSA (20 µg/mL), (**D**) LPS + NSA (20 µg/mL). Scale bar = 50 µm.

Phase-contrast microscopy was used to examine macrophage morphology after treatment Figure 9. Untreated (Figure 9A) and LPS-stimulated (Figure 9B) cultures retained predominantly adherent, well-spread cells, whereas exposure to SSA (Figure 9C) or NSA (Figure 9D) at 20 µg/mL was accompanied by increased cell rounding, reduced spreading and lower adherent density, in agreement with the reduced viability recorded at higher concentrations in the MTT assay.

### 3.12. Macrophage-Derived CD9^+^ EV Secretion

CD9^+^ EVs isolated by qEV size-exclusion chromatography and quantified by NanoFCM showed a median diameter of 75–79 nm across all conditions Figure 9, consistent with the expected exosome size range, whereas the particle-free buffer blank produced no detectable events, confirming assay specificity. LPS stimulation increased EV secretion approximately two-fold relative to untreated cells (1.77 × 10^9^ vs. 8.73 × 10^8^ particles/mL). Both salicylic acids were associated with a lower CD9^+^ EV concentration in the conditioned medium relative to LPS: with SSA, the concentration was 72% (4.92 × 10^8^ particles/mL, below the untreated control level), while NSA reduced it by 40% (1.07 × 10^9^ particles/mL). The EV size distribution was essentially unchanged across conditions, indicating that the compounds modulated the quantity rather than the size of the secreted vesicles. As EVs were quantified with a CD9-targeted approach in conditioned medium without normalization to cell number, and treatments affected morphology, this may reflect reduced release or fewer adherent cells.

Total protein in the isolated EV fractions was low across all conditions (3–60 µg/mL; Table 3), consistent with the removal of free protein by size-exclusion chromatography and supporting the purity of the qEV preparations.

**Figure 10:**
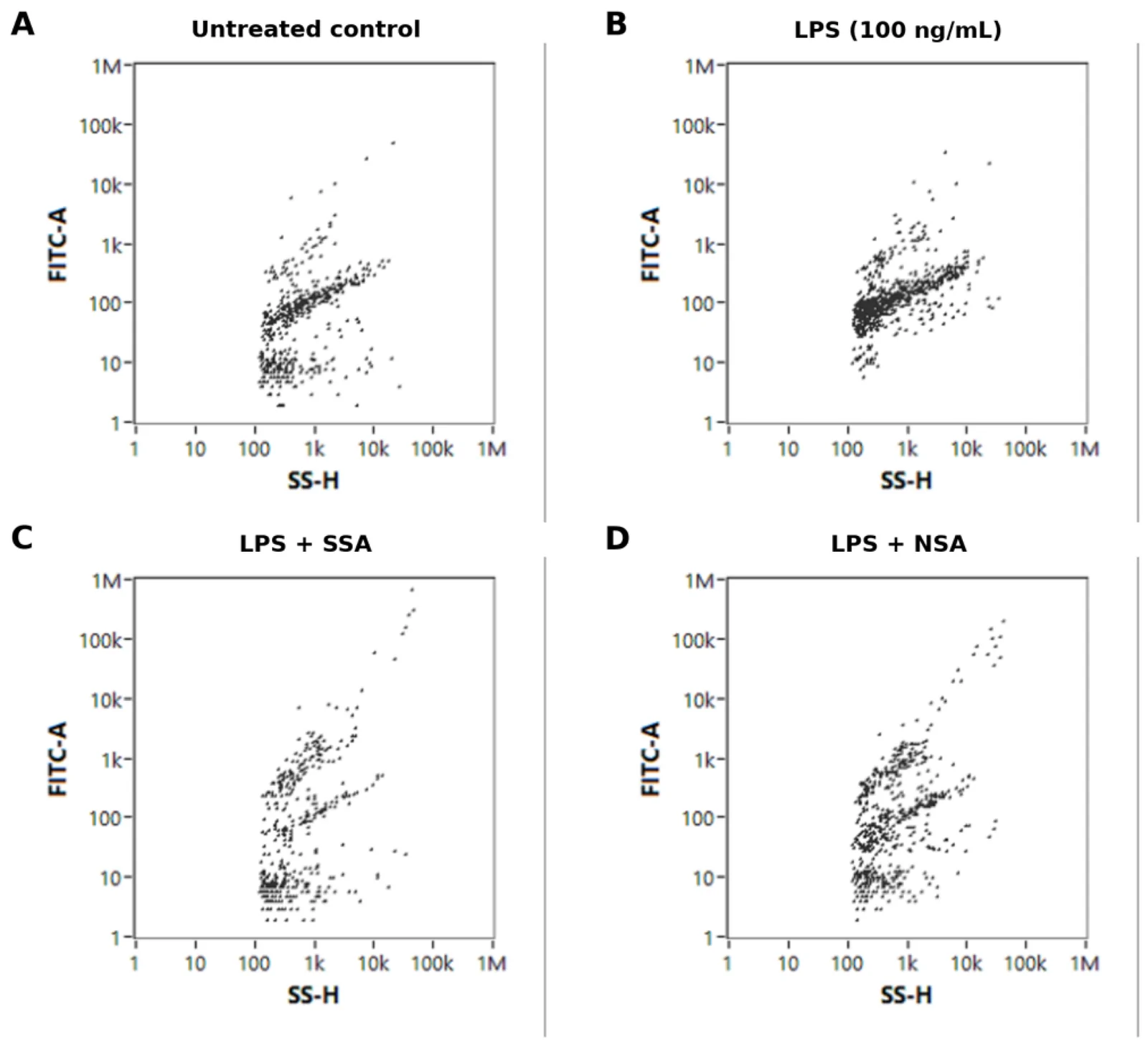
NanoFCM single-particle fluorescence scatter plots (FITC-A vs. SS-H) of EvoBrite CD9-labelled macrophage EVs: (**A**) untreated control, (**B**) LPS (100 ng/mL), (**C**) LPS + SSA, (**D**) LPS + NSA.

Single-particle fluorescence analysis (FITC-A versus side-scatter) confirmed CD9 (EvoBrite) labelling of the isolated vesicles across all conditions, verifying their identity as CD9^+^ EVs Figure 10.

### 3.13. Anti-Inflammatory Gene Expression

**Figure 11:**
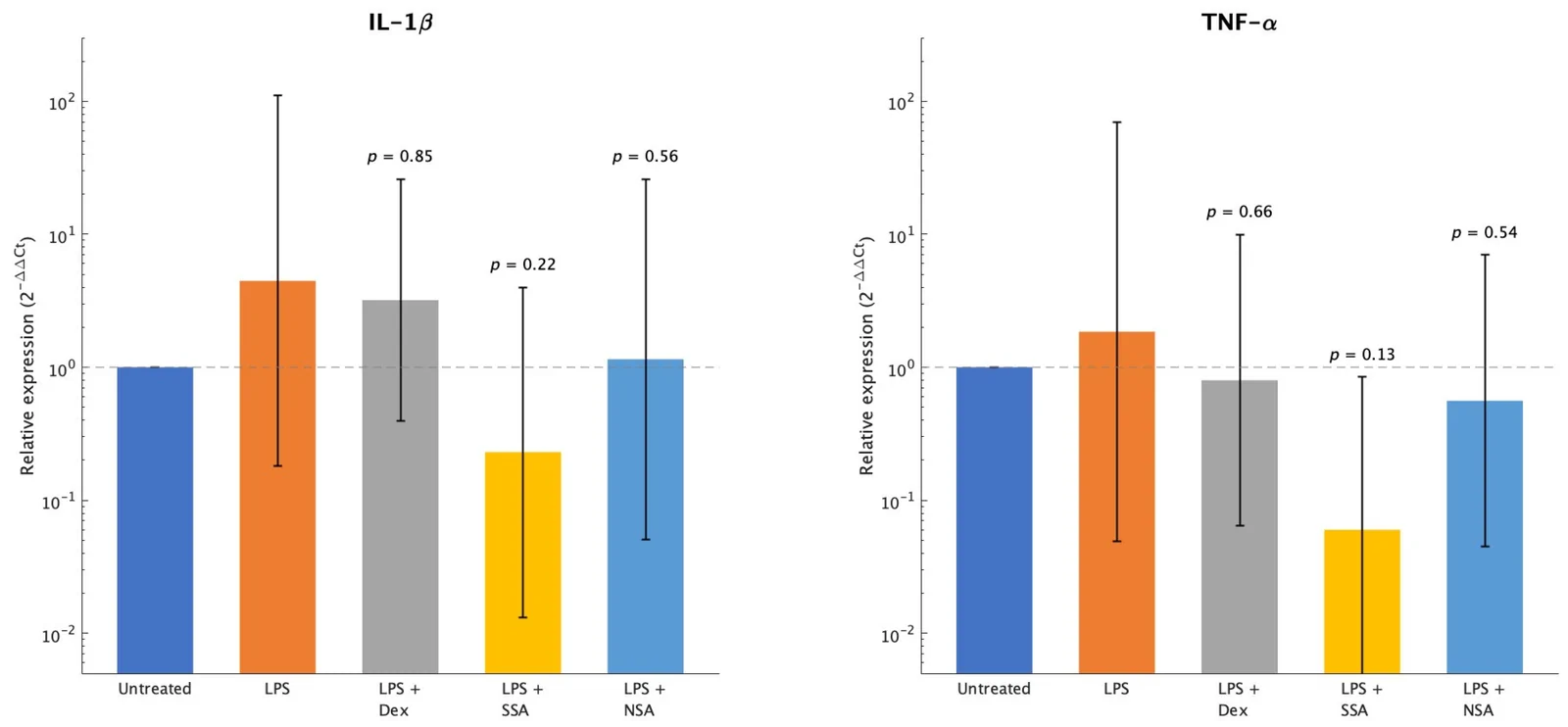
Relative expression of IL-1β and TNF-α in LPS-stimulated RAW 264.7 macrophages measured by RT-qPCR (2^−ΔΔCt^, GAPDH-normalized, calibrated to untreated cells. All treatments were applied in the presence of LPS (100 ng/mL): dexamethasone at 100 nM and SSA/NSA at 20 µg/mL (145 µM). Bars show the mean of three biological replicates (n = 3) and open circles the individual replicate values; the dashed line indicates no change versus untreated. *p*-values above the bars are from two-sample Student’s *t*-tests on ΔCt values (treatments versus LPS; LPS versus untreated); with n = 3.

The influence of salicylic acid on macrophage pro-inflammatory signalling was examined by RT-qPCR for the cytokine genes Il1b (IL-1β) and Tnf (TNF-α), normalized to GAPDH and expressed relative to untreated cells (2^−ΔΔCt^ method; n = 3 biological replicates; all treatments applied together with LPS at 100 ng/mL, with SSA and NSA at 20 µg/mL (145 µM) and dexamethasone at 100 nM). LPS stimulation increased both transcripts relative to untreated controls (IL-1β ≈ 9.9-fold; TNF-α ≈ 2.5-fold), confirming macrophage activation. The reference anti-inflammatory glucocorticoid dexamethasone (Dex + LPS) attenuated this induction (IL-1β 3.2-fold; TNF-α 0.78-fold relative to untreated). Both salicylic acids showed a similar or stronger apparent suppression: synthetic SA (SSA; shown as SS + LPS) reduced IL-1β to 0.23-fold and TNF-α to 0.06-fold of the untreated level, while natural SA (NSA; shown as NAS + LPS) reduced IL-1β to 1.13-fold and TNF-α to 0.56-fold. SSA showed the lowest transcript levels of the treated groups for both cytokines, the same rank order observed for CD9^+^ EV release. Two-sample *t*-tests on the ΔCt values, now with the full complement of three biological replicates, showed no statistically significant differences between groups: neither the LPS-induced increases over untreated cells (IL-1β *p* = 0.46; TNF-α *p* = 0.79) nor the suppression by any treatment relative to the LPS group reached significance (all *p* > 0.05; for TNF-α, SSA *p* = 0.14 and NSA *p* = 0.54; dexamethasone *p* = 0.66; all IL-1β comparisons *p* > 0.2). The apparent fold-changes are accompanied by wide between-replicate variability, and the differences described above should therefore be regarded as trends rather than established effects Figure 11.

### 3.14. Antioxidant Activity and Cytoprotection

**Figure 12:**
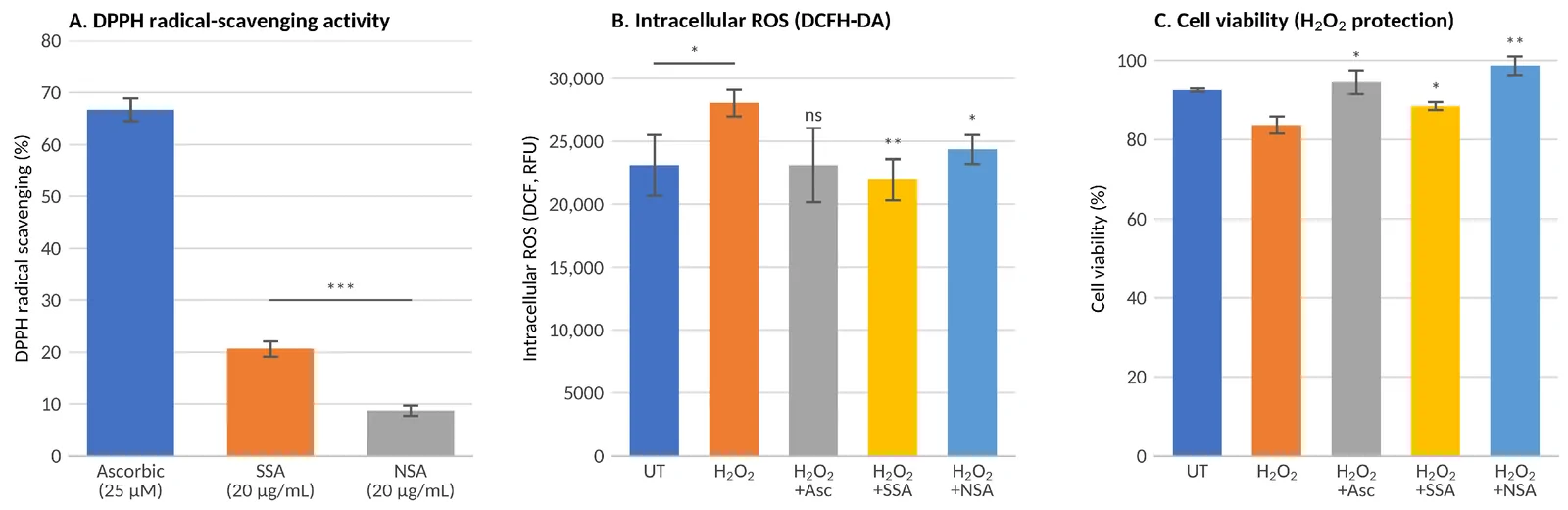
Antioxidant activity of natural (NSA) and synthetic (SSA) salicylic acid. (**A**) DPPH radical-scavenging activity of compound solutions (cell-free), with ascorbic acid (25 µM) as positive control. (**B**) Intracellular ROS in RAW 264.7 macrophages measured by DCFH-DA after 1 h exposure to 100 µM H_2_O_2_, with or without co-treatment. (**C**) Cell viability under the same conditions. Salicylic acid and ascorbic acid were applied at 20 µg/mL and 25 µM, respectively. Data are mean ± SD (n = 3 for DPPH and ROS; n = 4 for viability). Significance versus the H_2_O_2_ group (**B**,**C**) or between SSA and NSA (**A**): ns = not significant, * *p* < 0.05, ** *p* < 0.01, *** *p* < 0.001. (Ascorbic acid served as a positive control and was not tested at an equimolar concentration).

The antioxidant behaviour of the two salicylic acid forms was assessed at three levels: intrinsic radical-scavenging capacity (cell-free DPPH assay), intracellular ROS generation under oxidative challenge (DCFH-DA), and protection of cell viability against H_2_O_2_-induced injury in RAW 264.7 macrophages Figure 12.

In the cell-free DPPH assay, ascorbic acid produced strong radical scavenging (66.7 ± 2.1%). Both salicylic acids showed measurable but weaker activity, consistent with the single phenolic hydroxyl of the salicylate structure, and the synthetic form was significantly more active than the natural form (SSA 20.7 ± 1.6% versus NSA 8.7 ± 1.0%; *p* = 0.0008; Figure 12A).

Exposure to 100 µM H_2_O_2_ for 1 h raised intracellular ROS relative to untreated cells (1.21-fold; *p* = 0.030), confirming induction of oxidative stress. Co-treatment lowered ROS relative to the H_2_O_2_ group, the synthetic form giving the largest reduction (H_2_O_2_ + SSA −21.6%, *p* = 0.006; H_2_O_2_ + NSA −13.3%, *p* = 0.014; ascorbic acid −17.6%, *p* = 0.052; Figure 12B). Among the salicylic acids, the synthetic form again ranked above the natural form, matching the DPPH assay; ascorbic acid, however, no longer stood out from the salicylic acids in the cellular setting, in contrast to its clear superiority in the cell-free DPPH measurement.

These effects were paralleled at the level of cell viability. H_2_O_2_ reduced viability to 83.6 ± 2.1% of control, and all three antioxidants restored it significantly: the natural form was the most protective (H_2_O_2_ + NSA 98.7 ± 2.3%, *p* = 0.001), followed by ascorbic acid (94.4 ± 3.0%, *p* = 0.011) and the synthetic form (H_2_O_2_ + SSA 88.3 ± 1.0%, *p* = 0.016; Figure 12C). The two readouts are complementary: SSA was the stronger direct radical scavenger, whereas NSA afforded the greater cytoprotection, suggesting that the intrinsic scavenging capacity of the molecule and its protective effect within cells are governed by partly distinct factors, plausibly reflecting the differences in solid-state properties and cellular availability between the two forms.

A note on the ascorbic acid comparison. In the cell-free DPPH assay, ascorbic acid was, as expected, the strongest radical scavenger, whereas in the cellular assays (DCFH-DA and viability) the three compounds afforded comparable protection. This apparent discrepancy is consistent with the biology of the macrophage model: cellular uptake of ascorbic acid depends on sodium-dependent vitamin C transporters (SVCT1/2), which are expressed at limited and variable levels in RAW 264.7 macrophages, so extracellular ascorbate is not fully reflected in intracellular activity [61,62]. Macrophages also possess a high endogenous antioxidant capacity (glutathione, catalase, superoxide dismutase), which reduces the relative contribution of an added antioxidant, and ascorbate can behave as a pro-oxidant intracellularly in the presence of transition metals [63]. The salicylic acids, being lipophilic, cross the plasma membrane by passive diffusion and are therefore less dependent on transporter-mediated uptake, which may support their intracellular effects. The intrinsic (DPPH) and cellular measures of antioxidant activity thus need not follow the same rank order, mirroring the dissociation between solubility and permeation observed for the two forms elsewhere in this study.

## 4. Discussion

The synthesis route of SA has a marked effect on its solid-state properties that are responsible for its dissolution behaviour and transdermal permeation performance, as demonstrated in this study. The same molecular structure of NSA and SSA exhibited substantial differences in crystal morphology, crystallinity, and biopharmaceutical properties, indicating that solid-state modifications are crucial in determining pharmaceutical functionality [11,64].

From a physicochemical perspective, a well-ordered crystal lattice with few structural defects is generally associated with a higher lattice energy, which can limit solubility by strengthening intermolecular interactions. Such structural order is typically associated with a higher lattice energy, which limits solubility by strengthening intermolecular interactions within the crystal structure [9,47]. However, this study found no significant difference in solubility between SSA and NSA (2.239 vs. 2.115 mg/mL; *p* > 0.05), indicating that the two forms have comparable equilibrium solubility. This suggests that, for these materials, equilibrium solubility is not strongly governed by the differences in crystal habit observed between them. The uniform, needle-like morphology of SSA may instead be more relevant to its early dissolution behaviour than to its equilibrium solubility [9,65,66].

Although NSA and SSA show only minor differences in their XRD patterns, both forms display sharp, well-defined reflections consistent with crystalline salicylic acid, as confirmed by comparison with the simulated reference pattern (CSD refcode SALIAC). The differences between the two forms are therefore better explained by variations in crystal habit and preferred orientation than by the presence of an amorphous phase [67,68]. These structural features may increase molecular mobility and could potentially influence membrane transport. Although NSA showed a numerical tendency toward higher steady-state flux and permeability coefficient, the present findings do not establish a direct causal relationship between the solid-state characteristics of two compounds and transdermal permeability [69,70,71].

The correlation found between dissolution and permeation in this study is a significant biopharmaceutical problem. SSA showed fast initial dissolution and higher cumulative permeation, whereas NSA showed numerically higher steady-state flux and permeability coefficient together with a shorter lag time. This apparent discrepancy suggests that transdermal performance may not be determined solely by solubility [72,73]. According to Fick’s first law of diffusion, flux is influenced by the concentration gradient, diffusion coefficient, and partitioning behaviour of the drug within the membrane [74]. However, given the variability observed in the permeation parameters, these differences should be interpreted as trends rather than definitive differences in permeation performance.

The tendency toward higher permeability observed for NSA may be related to differences in crystal habit and particle characteristics between the two forms, which could increase molecular mobility and potentially facilitate partitioning into the *stratum corneum* [75,76]. The *stratum corneum* is a well-organized lipid layer that serves as the primary barrier to transdermal delivery systems. Molecules with greater flexibility and lipid-disrupting capability exhibit better permeation [77,78]. In this scenario, the structural diversity and possible traces in the NSA formulation could disrupt lipid structure, thereby increasing diffusivity.

Additionally, the numerically shorter lag time observed for NSA may indicate a tendency toward more rapid establishment of permeation across the skin barrier. This observation may be consistent with previous reports suggesting that differences in solid-state characteristics can influence drug permeation behaviour [79]. Nevertheless, considering the variability of the measurements, this interpretation should be regarded as tentative.

The results suggest a relationship between dissolution rate and permeability which can be attributed to the structural properties of the drug in the solid state. SSA has a more homogeneous crystalline structure that may help drug dissolution. NSA differed from SSA in its solid-state characteristics and showed a tendency toward more favourable permeation parameters, although further studies with larger replicate numbers would be required to establish the significance of these differences. These findings highlight that, in drug development, the selection of the solid-state form should be based not only on physicochemical properties but also on the intended route of administration and therapeutic purpose. In addition, the naturally derived route used for NSA may offer sustainability advantages, although these remain to be quantified. Integrating green-chemistry approaches with solid-state engineering could contribute to the development of more efficient drug delivery systems. It should be noted that, at the concentration used in the cellular assays [80] (20 µg/mL, well below the aqueous solubility of 2 mg/mL), both salicylic acid forms were fully dissolved. The original crystalline lattice is therefore no longer present under these conditions, and the differences observed in the antioxidant, extracellular-vesicle and gene-expression assays cannot be attributed to the solid-state form of the two materials; the solid-state characterization and the cellular assays are presented as complementary rather than causally linked. The cellular experiments extend this physicochemical comparison into a biological context. Despite sharing an identical molecular structure, SSA was modestly more cytotoxic to macrophages than NSA (IC_50_ 74.7 vs. 145.6 µg/mL), consistent with subtle differences in solid-state behaviour between the two materials; nonetheless, both compounds were well tolerated within the low-concentration range relevant to topical use. Importantly, both salicylic acids were associated with a lower CD9^+^ EV concentration in the conditioned medium relative to the LPS-induced cells, with SSA being more effective than NSA. Because macrophage-derived EVs act as carriers of pro-inflammatory signals, this reduction in CD9^+^ EV secretion provides a functional, cell-level correlate of the anti-inflammatory activity classically attributed to salicylic acid, and links the observed biological response to a measurable biological response.

From an immunopharmacological perspective, macrophage-derived extracellular vesicles are increasingly recognized as active mediators of inflammation that shuttle cytokines, microRNAs and tetraspanins such as CD9 between cells; agents that limit their release may therefore attenuate inflammatory amplification beyond the direct inhibition of prostaglandin synthesis [81,82]. The ability of both salicylic acid forms to lower LPS-induced CD9^+^ EV output, together with their favourable macrophage biocompatibility, positions them as candidate immunomodulators for inflammatory skin conditions, and the greater effect of SSA indicates that particle characteristics can fine-tune this activity. Consistent with this mechanism, RT-qPCR analysis showed that there was no statistical difference between the two salicylic acids compared to the LPS-induced transcription of IL-1β and TNF-α.

Several considerations, however, argue against interpreting these RT-qPCR values as a direct measure of anti-inflammatory potency. First, with the full set of three biological replicates, none of the between-group differences reached statistical significance, including the LPS-induced increase over untreated cells (IL-1β *p* = 0.46; TNF-α *p* = 0.79) and the reductions by each treatment relative to the LPS group (TNF-α: SSA *p* = 0.14, NSA *p* = 0.54, dexamethasone *p* = 0.66; all IL-1β comparisons *p* > 0.2). The apparent fold-changes are accompanied by wide between-replicate variability and are therefore best read as trends. Second, the concentration applied (20 µg/mL, 145 µM) lies close to the measured cytotoxicity thresholds: it is 3.7-fold below the IC_50_ of SSA (74.7 µg/mL) and only 1.6-fold above the lower bound of its 95% confidence interval (31.3 µg/mL), and the same exposure was accompanied by cell rounding and reduced adherence in the morphological analysis (Section 3.7); a partial contribution of reduced viability to the apparent transcript reduction can therefore not be excluded. Third, 145 µM lies well below the range at which salicylate is known to act on this pathway: inhibition of IKKβ and downstream NF-κB signalling by salicylate is typically observed only at low-millimolar concentrations (1–5 mM) [34,35], which also exceed the 1–2 mM plasma levels reached [83] during high-dose salicylate therapy, so a pronounced, mechanism-based suppression would not be anticipated at the concentration used here [84].

A further consideration concerns the relationship between the effective and the cytotoxic concentration ranges. Salicylate-mediated inhibition of NF-κB signalling is typically observed only at low-millimolar concentrations, which lie within the cytotoxic range of both compounds measured (IC_50_ = 74.7 µg/mL for SSA and 145.6 µg/mL for NSA). In this macrophage model, there is therefore little separation between the concentration needed for mechanism-based cytokine suppression and that at which viability is compromised. Consistent with this, at the low, sub-cytotoxic concentration actually used in our assays (20 µg/mL), salicylic acid did not significantly suppress the classical M1 transcriptional programme (IL-1β, TNF-α), and the transcriptional readouts are therefore best regarded as supportive rather than quantitative. Both forms nonetheless reduced LPS-induced CD9^+^ EV release (Section 3.9), suggesting that extracellular-vesicle output may provide a more sensitive readout of macrophage modulation than transcriptional endpoints, and one that is at least partly independent of the classical NF-κB–driven M1 pathway. A full characterization of M1/M2 polarization, including dedicated M2 markers (for example Arg-1, CD206, IL-10), was beyond the scope of the present study and represents an important direction for future work.

The transcriptional results are therefore best read as consistent in direction with the CD9^+^ EV data rather than as an independent, quantitative confirmation of anti-inflammatory potency. A definitive assessment would require the reference-gene signal to be restored to its normal range, confirmation of adequate LPS induction, a salicylate concentration series extending toward the millimolar range with a viability readout acquired on the same plates, and the full complement of biological replicates (n = 3). Across the cellular readouts, the synthetic form was consistently the more active of the two salicylic acids. Relative to the LPS-stimulated group, it lowered CD9^+^ EV output to 27.8% versus 60.5% for the natural form, reduced intracellular ROS by 21.6% versus 13.3%, scavenged DPPH radicals more than twice as effectively (20.7% versus 8.7%), and produced stronger suppression of IL-1β (0.23- versus 1.13-fold) and TNF-α (0.06- versus 0.56-fold) transcripts. The only exception was protection of cell viability against H_2_O_2_-induced injury, where the natural form was the more protective (98.7% versus 88.3%). Because all treatments were applied at a single sub-cytotoxic concentration (20 µg/mL), these endpoints describe a consistent rank order across several biological levels rather than a formal dose–response correlation.

Both forms lowered H_2_O_2_-induced intracellular ROS and preserved viability, but the natural form afforded the greater cytoprotection despite being the weaker chemical scavenger. This dissociation between intrinsic and cellular antioxidant activity parallels the dissociation between solubility and permeation reported above and reinforces the central theme of this study: the biological behaviour of salicylic acid is governed not only by its molecular structure but by the solid-state and physicochemical properties that determine its availability within a biological system. The behaviour of the ascorbic acid control merits comment. Although ascorbic acid was, as expected, by far the strongest scavenger in the cell-free DPPH assay, it did not stand out from the salicylic acids in the cellular assays. This is consistent with the biology of the macrophage model. Cellular uptake of ascorbate is not passive but depends on the sodium-dependent vitamin C transporter SVCT2, whose expression is regulated during macrophage differentiation and activation, so that extracellular ascorbate is not fully reflected in intracellular activity [70,71]. Macrophages additionally maintain a high endogenous antioxidant capacity, which reduces the relative contribution of an added antioxidant [72]. Moreover, in the presence of H_2_O_2_ and the transition-metal traces present in culture medium, ascorbate can itself act as a pro-oxidant through Fenton chemistry, further limiting its net protective effect in a cellular setting [73]. The salicylic acids, being lipophilic, cross the plasma membrane by passive diffusion and are less dependent on transporter-mediated uptake, which may support their intracellular effects. The intrinsic (DPPH) and cellular measures of antioxidant activity therefore need not follow the same rank order. Taken together, the cellular endpoints follow a consistent pattern. Across chemical radical scavenging (DPPH), intracellular ROS suppression, downregulation of IL-1β and TNF-α transcripts, and reduction in CD9^+^ EV release, the synthetic form was the more active of the two salicylic acids: relative to the LPS-stimulated group, it lowered EV output for the natural form, reduced ROS by 21.6% versus 13.3%, and scavenged DPPH radicals more than twice as effectively. Because all treatments were applied at a single sub-cytotoxic concentration (20 µg/mL), chosen so that the extracellular-vesicle, transcriptional and antioxidant readouts could be compared under matched conditions, these endpoints are not independent dose–response measurements and a formal quantitative correlation is not claimed; rather, they describe the same rank order across several biological levels. The one exception was protection of cell viability against H_2_O_2_-induced injury, where the natural form was the more protective. This divergence is consistent with the interpretation above: the synthetic form is the stronger intrinsic radical scavenger and the more active modulator of macrophage inflammatory output, whereas the greater cytoprotection of the natural form may reflect its distinct solid-state properties and cellular availability. The convergence of four functional readouts on the same rank order nonetheless supports the central conclusion that the synthesis route, through its effect on crystal structure, governs the physical (dissolution and permeation) behaviour of salicylic acid across multiple endpoints rather than at a single one. SSA and NSA showed similar dissolution characteristics, whereas NSA exhibited a trend toward higher flux and permeability coefficient, together with the advantage of a more sustainable production route. These findings underscore the importance of the production method and crystalline structure in influencing the pharmaceutical performance of salicylic acid. Beyond these physicochemical differences, both forms were non-cytotoxic to RAW 264.7 macrophages and associated with a lower CD9^+^ EV concentration in the conditioned medium compared to LPS-induced CD9^+^ extracellular-vesicle release, revealing an immunomodulatory dimension of salicylic acid relevant to its topical anti-inflammatory use. Table 4 shows the overall comparison of SSA and NSA results.

## 5. Conclusions

It should be noted that the findings from the present investigation suggest the significant impact of the way the salicylic acid was synthesized on its physicochemical properties and biopharmaceutical efficiency. The two types of salicylic acid—natural and synthetic, having similar structures but different origins—showed notable differences in physical form, together with numerical differences in skin permeation parameters and dissolution characteristics.

Namely, the synthetic type of salicylic acid, characterized by its highly ordered crystalline structure and homogeneous morphology, demonstrated comparable equilibrium solubility with fast initial dissolution, which could make it appropriate for fast-release formulations with the advantage of its stable state. In contrast, the less-ordered NSA showed numerically higher steady-state flux and permeability coefficient together with a shorter lag time. These findings suggest a tendency toward more favourable early permeation characteristics for NSA, although the observed variability warrants cautious interpretation.

This research illustrates the basic compromise existing between dissolution and permeation and associated with solid-state properties. Notably, the findings indicate that solubility does not play the only role in determining drug effectiveness when using transdermal administration, where diffusion becomes equally important along with membrane partitioning.

At the same time, the employment of green synthesis methods in obtaining naturally derived salicylic acid represents an additional benefit of sustainable production of drugs that corresponds to modern tendencies in developing potentially more sustainable production, which remains to be quantified.

In general, the study shows how crucial the choice of the solid-state form of the drug molecule could be in terms of its effectiveness and indicates that such choice should be made depending on the desired way of administration and therapeutic purpose.

The biological evaluation reinforced these conclusions. Both salicylic acids were non-cytotoxic to RAW 264.7 macrophages across the low-concentration range relevant to topical use, with SSA showing a modestly lower IC_50_ (74.7 µg/mL) than NSA (145.6 µg/mL). Importantly, both forms suppressed the LPS-induced secretion of CD9^+^ macrophage extracellular vesicles (a marker of macrophage activation), with SSA being the more effective, thereby linking the biological response between the two materials to a measurable anti-inflammatory response. Together, these findings indicate that the choice between natural and synthetic salicylic acid influences not only physicochemical performance but also cellular biocompatibility and immunomodulatory behaviour. Consistent in direction with the EV data, RT-qPCR analysis showed no difference between forms in the LPS-induced expression of TNF-α.

The antioxidant characterization extended this comparison. In the cell-free DPPH assay, the synthetic form was the stronger radical scavenger (20.7% versus 8.7% for the natural form), and it likewise produced the larger reduction in H_2_O_2_-induced intracellular ROS, whereas the natural form afforded the greater protection of cell viability. This dissociation between intrinsic chemical scavenging and cellular cytoprotection mirrors the dissociation observed between solubility and permeation, underlining that the solid-state form governs the physical (dissolution and permeation) behaviour across multiple, mechanistically distinct endpoints rather than at a single one.

Together, these findings indicate that the choice between natural and synthetic salicylic acid influences not only physicochemical performance but also antioxidant capacity, cellular biocompatibility and immunomodulatory behaviour, providing a rational basis for selecting the appropriate form according to the intended cosmetic or dermatological application.

## Figures and Tables

**Table 1:** Solubility values obtained from HPLC analyses of NSA and SSA.

	Natural Salicylic Acid (Water Solubility)	Synthetic Salicylic Acid (Water Solubility)	Natural Salicylic Acid (Res. Avg. Solubility)	Synthetic Salicylic Acid (Res. Avg. Solubility)
	2.098	2.267	4.804	5.572
	2.116	2.272	4.816	4.895
	2.131	2.177	4.811	4.554
Mean	2.115	2.239	4.810	5.007
±SD	0.017	0.054	0.006	0.518

Data are presented as mean ± SD (n = 3).

**Table 2:** Permeation parameters of NSA and SSA.

	Steady State Flux (µg·cm^−2^·hour^−1^)	Permeability Coefficient (cm·hour^−1^)	Lag Time (Hour)
Natural Salicylic Acid	39.94 ± 16.43	15.98 ± 6.57 × 10^−3^	2.47 ± 1.71
Synthetic Salicylic Acid	35.57 ± 13.91	14.23 ± 5.56 × 10^−3^	3.28 ± 0.55

Values are expressed as mean ± SD (n = 3).

**Table 3:** Total protein content and particle-to-protein ratio of isolated macrophage EV fractions.

Condition	Median Size (nm)	Mean Size (nm)	Concentration (Particles/mL)
Untreated control	74.8	80.1 ± 19.1	8.73 × 10^8^ ± 1.7 × 10^8^
LPS (100 ng/mL)	75.2	82.1 ± 23.8	1.77 × 10^9^ ± 2.7 × 10^8^
LPS + SSA	79.2	82.0 ± 23.7	4.92 × 10^8^ ± 1.0 × 10^8^
LPS + NSA	78.2	83.1 ± 22.9	1.07 × 10^9^ ± 1.6 × 10^8^

**Table 4:** Overall comparison of SSA and NSA.

Readout	SSA (Synthetic)	NSA (Natural)	More Active
DPPH radical scavenging (%)	20.7 ± 1.6	8.7 ± 1.0	SSA
Intracellular ROS reduction vs. H_2_O_2_ (%)	21.6 ± 5.9	13.3 ± 4.1	SSA
CD9^+^ EV concentration (% of LPS)	27.8 ± 7.1	60.5 ± 12.9	SSA
IL-1β expression (fold vs. untreated)	0.23	1.13	SSA
TNF-α expression (fold vs. untreated)	0.06	0.56	SSA
Viability protection vs. H_2_O_2_ (%)	88.3 ± 1.0	98.7 ± 2.3	NSA

## Data Availability

The original contributions presented in this study are included in the article/Supplementary Material. Further inquiries can be directed to the corresponding author.

## Supplementary Materials

The following supporting information can be downloaded at: https://www.mdpi.com/article/10.3390/antiox15101287/s1, Figure S1: ^1^H and ^13^C NMR spectra of synthetic (SSA; a, c) and naturally derived (NSA; b, d) salicylic acid (Bruker, 500 MHz, DMSO-d_6_); Figure S2: GC-MS chromatogram of the naturally derived salicylic acid (NSA, Plantraction WG-99); Figure S3: GC-MS chromatogram of the synthetic salicylic acid (SSA) prepared in-house.
