# Supplementary material for: Naturally Derived Versus Synthetic Salicylic Acid: Physicochemical Properties, Transdermal Permeation and Macrophage Immunomodulation
Source: Antioxidants (Basel). 2026 Oct 3;15(10):1287. doi: 10.3390/antiox15101287 (PMC13700645; doi:10.3390/antiox15101287)
Supplement: Supplementary file 1 [file antioxidants-15-01287-s001.zip › antioxidants-4512620-supplementary.pdf]

Supplementary Material

Naturally Derived versus Synthetic Salicylic Acid: Physicochemical Properties, Transdermal Permeation and Macrophage Immunomodulation

Manuscript ID: antioxidants-4512620

This Supplementary Material provides the spectroscopic (NMR) and chromatographic (GC-MS) characterization of the naturally derived (NSA) and synthetic (SSA) salicylic acid used in this study, confirming their chemical identity and purity. Representative chromatograms and spectra are provided below, and the corresponding acquisition details have been added to the Methods of the main manuscript.

1. NMR spectroscopy

The identity of both forms was confirmed by ^{1}H and ^{13}C NMR spectroscopy (Bruker, 500 MHz, DMSO-d_6). The spectra of NSA and SSA are essentially superimposable and are fully consistent with the structure of salicylic acid.

^{1}H NMR (500 MHz, DMSO-d_6): $\delta \approx 11.5$ (OH), 7.79 (dd, 1H), 7.40–7.50 (m, 1H), 6.90 (m, 1H), 6.80 (m, 1H); the carboxylic acid proton was additionally observed for SSA at ≈ 13.1 ppm.

^{13}C NMR (500 MHz, DMSO-d_6): $\delta \approx 172.6$ (COOH), 161.8 (C–OH), 135.8, 130.6, 119.4 and 117 (aromatic carbons).

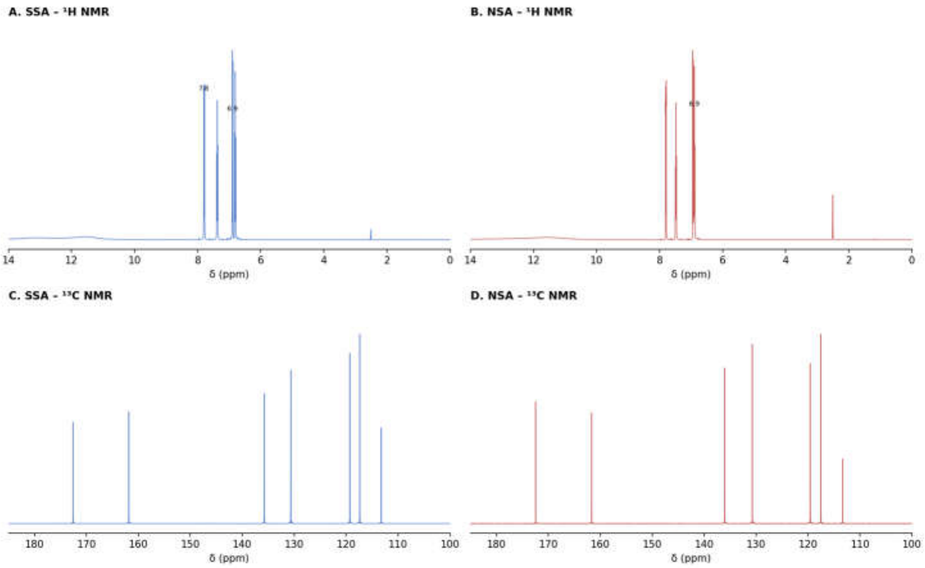

Figure S1. ^{1}H and ^{13}C NMR spectra of synthetic (SSA; a, c) and naturally derived (NSA; b, d) salicylic acid (Bruker, 500 MHz, DMSO-d_6). The spectra of the two forms are essentially identical and consistent with salicylic acid.

2. GC-MS analysis

The purity of the two materials was determined by GC-MS (area %). NSA (Plantraction WG-99, obtained from wintergreen oil by a green-chemistry route) gave a single salicylic acid peak with an assay of $\geq 99\%$ (area %) and no detectable phenol, 4-hydroxybenzoic acid or 4-hydroxyisophthalic acid (Figure S2). SSA, prepared in-house by the Kolbe–Schmitt reaction,

showed the salicylic acid peak as the principal component with an assay of approximately 93% (area %), together with minor additional peaks attributable to residual by-products of the synthetic route (Figure S3).

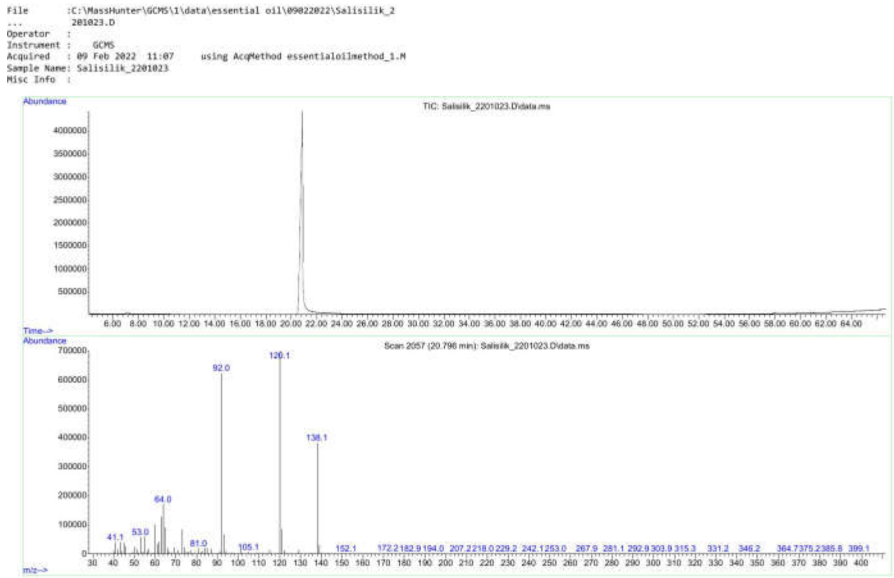

Figure S2. GC-MS chromatogram of the naturally derived salicylic acid (NSA, Plantraction WG-99): a single salicylic acid peak (≥ 99% area; characteristic MS fragments m/z 138, 120, 92, 64, 53).

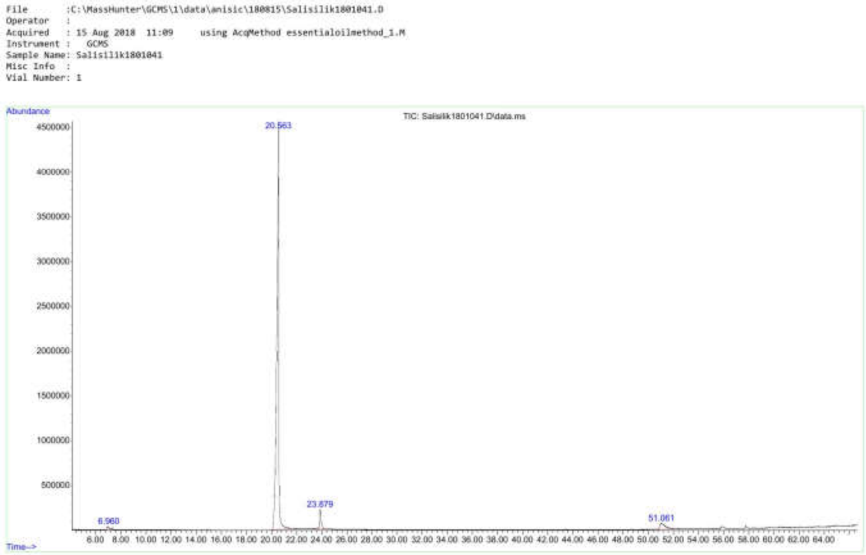

Figure S3. GC-MS chromatogram of the synthetic salicylic acid (SSA) prepared in-house: salicylic acid as the principal peak (≈ 93% area) with minor additional peaks corresponding to residual synthesis by-products.
